# Supplementary material for: Plasmodium vivax latent liver infection is characterized by persistent hypnozoites, hypnozoite-derived schizonts, and time-dependent efficacy of primaquine
Source: Mol Ther Methods Clin Dev. 2022 Aug 2;26:427–40. doi: 10.1016/j.omtm.2022.07.016 (PMC9418049; doi:10.1016/j.omtm.2022.07.016)
Supplement: Document S1. Figures S1–S4 [file mmc1.pdf]

OMTM, Volume *26*

Supplemental information

Plasmodium vivax latent liver infection is characterized by persistent hypnozoites, hypnozoite-derived schizonts, and time-dependent efficacy of primaquine

Erika L. Flannery, Niwat Kangwanrangsan, Vorada Chuenchob, Wanlapa Roobsoong, Matthew Fishbaugher, Kevin Zhou, Zachary P. Billman, Thomas Martinson, Tayla M. Olsen, Carola Schäfer, Brice Campo, Sean C. Murphy, Sebastian A. Mikolajczak, Stefan H.I. Kappe, and Jetsumon Sattabongkot

Supplemental Material

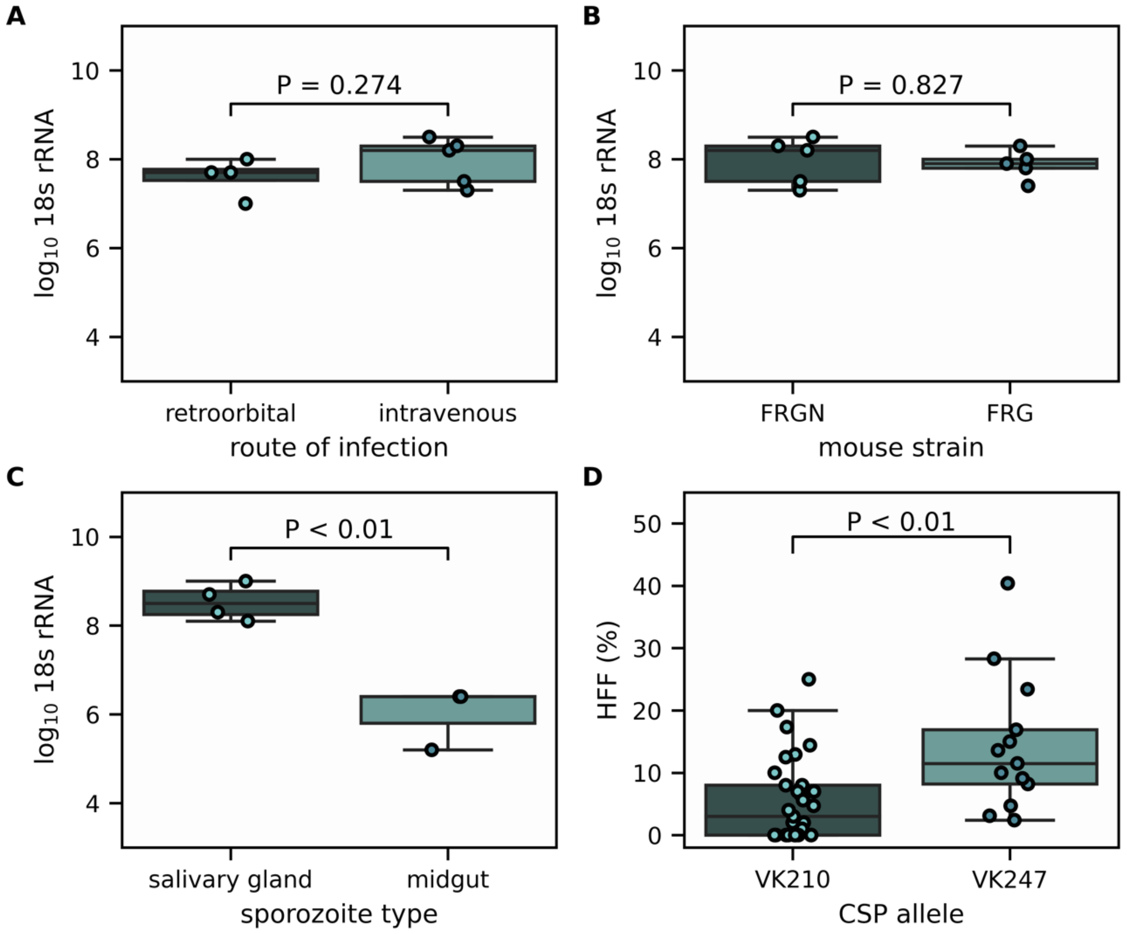

Figure S1. Additional characteristics of *P. vivax* liver-chimeric humanized mouse infections. (A) *P. vivax* 18S rRNA total liver burden 8 days after infection with 1.0 million sporozoites either intravenously or retro orbitally (p = 0.274, unpaired t-test). (B) *P. vivax* 18S rRNA total liver burden 8 days post-infection in FRGN (NOD) vs. FRG (C56Bl/6) mice infected with 1 million sporozoites (p = 0.827, unpaired t-test). (C) *P. vivax* 18S rRNA total liver burden 8 days post- infection with 0.6 million salivary gland- or midgut oocyst-dissected sporozoites (p < 0.01, unpaired t-test). (D) Hypnozoite formation frequency (HFF) of all isolates in Figure 1E-G (7 VK210 allele isolates and 3

VK247 allele isolates, the number of animals contributing from each isolate is denoted in Figure 1F, each dot represents one animal) by CSP allele type ($p < 0.01$, Mann Whitney test). In all figures, each data point represents observations from one animal. Parasite isolates used were (A, B): VUNL41, (C): VTTY111.

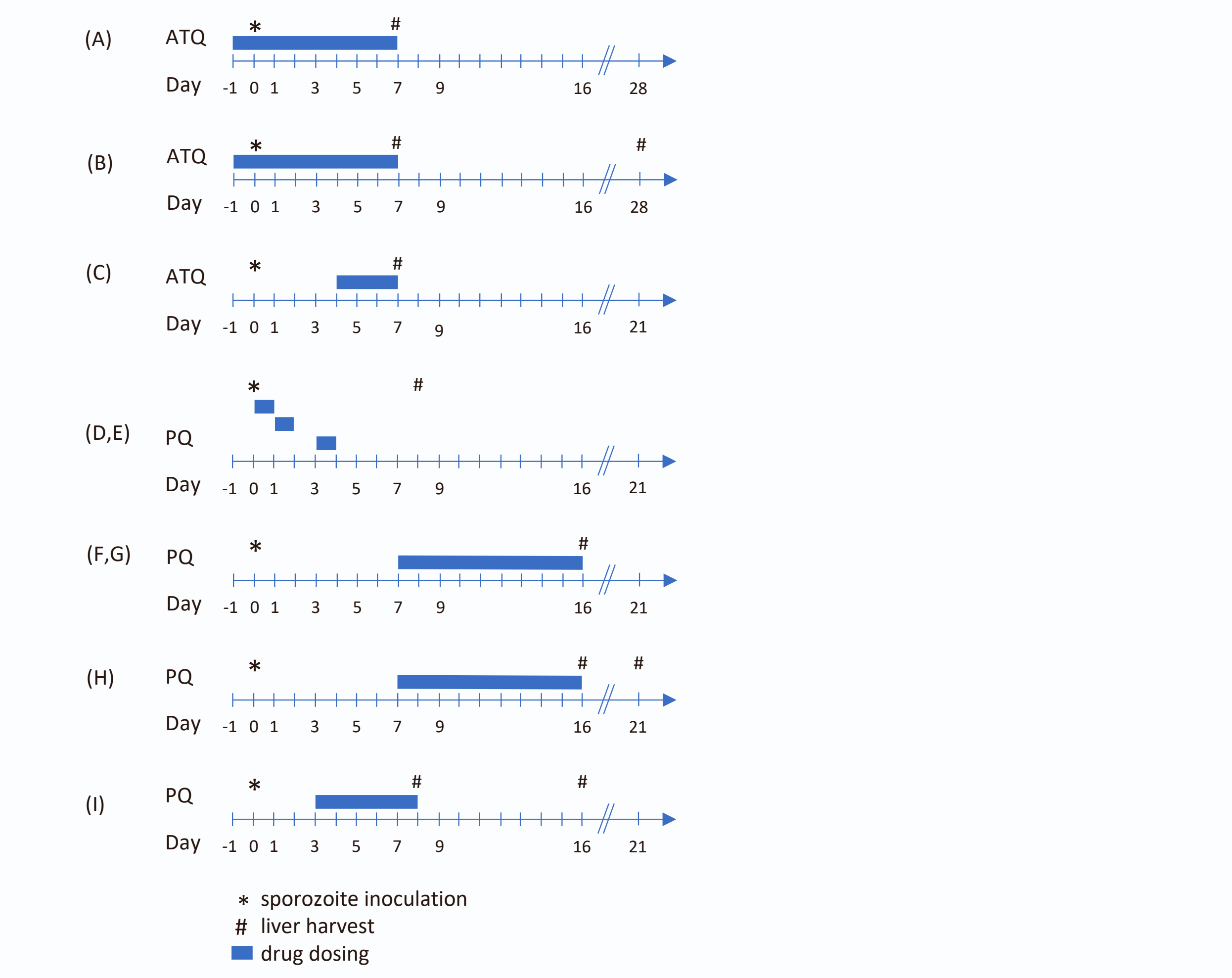

Figure S2. Dosing regimens for experiments with primaquine and atovaquone. FRG huHep mice were infected and treated at different timepoints with either atovaquone (ATQ) or primaquine (PQ). Results are presented in Figure 4.

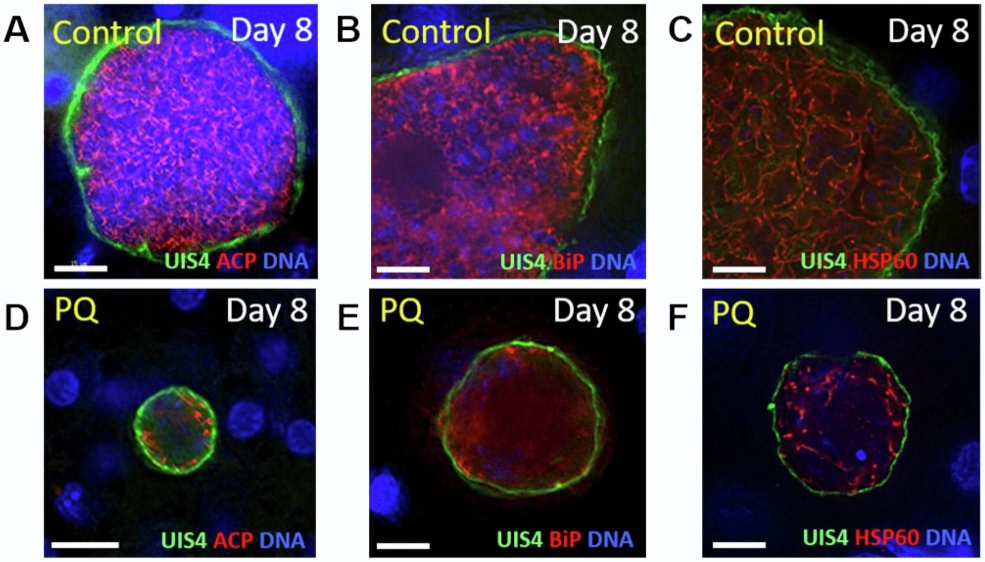

Figure S3. Effect of Primaquine treatment on *P. vivax* liver stage organelle development. FRG huHep mice infected with *P. vivax* sporozoites were untreated (Control) or treated with Primaquine (PQ) at day 3 and 4 and sacrificed at day 8 post inoculation. Immunofluorescence staining (IFA) was performed for (A,D) apicoplast (ACP – red), (B,E) ER (BiP – red), (C,F) mitochondria (HSP60 -red), parasitophorous vacuole membrane (UIS4 - green), and DNA (DAPI - blue); scale 10 μm.

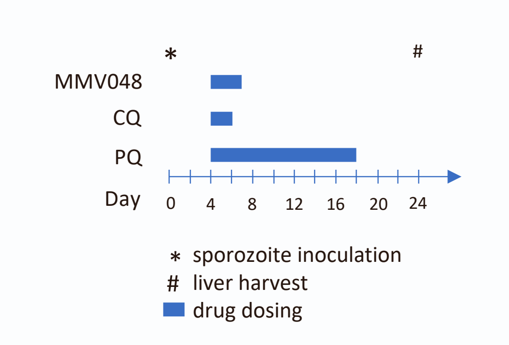

Figure S4. Dosing regimen used to demonstrate radical cure. FRG huHep mice were infected and livers were harvested 24 days later. Mice were either untreated, treated with MMV048 alone (30 mg/kg, days 4-7), PQ alone (30 mg/kg, days 4 -18), CQ alone (10 mg/kg, days 4-6) or in combination.
