# Supplementary material for: Plasmodium vivax latent liver infection is characterized by persistent hypnozoites, hypnozoite-derived schizonts, and time-dependent efficacy of primaquine
Source: Mol Ther Methods Clin Dev. 2022 Aug 2;26:427–40. doi: 10.1016/j.omtm.2022.07.016 (PMC9418049; doi:10.1016/j.omtm.2022.07.016)
Supplement: Document S2. Article plus supplemental information [file mmc2.pdf]

Molecular Therapy
Methods & Clinical Development
Original Article

Plasmodium vivax latent liver infection is characterized by persistent hypnozoites, hypnozoite-derived schizonts, and time-dependent efficacy of primaquine

Erika L. Flannery,[1,7] Niwat Kangwanrangsan,[2] Vorada Chuenchob,[1,7] Wanlapa Roobsoong,[3] Matthew Fishbaugher,[1,7] Kevin Zhou,[4] Zachary P. Billman,[4] Thomas Martinson,[1,8] Tayla M. Olsen,[4] Carola Schäfer,[1,9] Brice Campo,[5] Sean C. Murphy,[4] Sebastian A. Mikolajczak,[1,7] Stefan H.I. Kappe,[1,6] and Jetsumon Sattabongkot[3]

[1]Center for Global Infectious Disease Research, Seattle Children's Research Institute, Seattle, WA 98109, USA; [2]Department of Pathobiology, Faculty of Science, Mahidol University, Bangkok, Thailand; [3]Mahidol Vivax Research Unit, Faculty of Tropical Medicine, Mahidol University, Bangkok, Thailand; [4]Department of Laboratory Medicine and Pathology, and Department of Microbiology, University of Washington, Seattle, WA 98115, USA; [5]Medicines for Malaria Venture, Geneva, Switzerland; [6]Department of Pediatrics, University of Washington, Seattle, WA 98105, USA

Plasmodium vivax **is a malaria-causing pathogen that establishes a dormant form in the liver (the hypnozoite), which can activate weeks, months, or years after the primary infection to cause a relapse, characterized by secondary blood-stage infection. These asymptomatic and undetectable latent liver infections present a significant obstacle to the goal of global malaria eradication. We use a human liver-chimeric mouse model (FRG huHep) to study *P. vivax* hypnozoite latency and activation in an *in vivo* model system. Functional activation of hypnozoites and formation of secondary schizonts is demonstrated by first eliminating primary liver schizonts using a schizont-specific antimalarial tool compound, and then measuring recurrence of secondary liver schizonts in the tissue and an increase in parasite RNA within the liver. We also reveal that, while primaquine does not immediately eliminate hypnozoites from the liver, it arrests developing schizonts and prevents activation of hypnozoites, consistent with its clinical activity in humans. Our findings demonstrate that the FRG huHep model can be used to study the biology of *P. vivax* infection and latency and assess the activity of anti-relapse drugs.**

INTRODUCTION

The eradication of malaria remains a prominent public health goal. While *Plasmodium falciparum* infection contributes the greatest to malaria disease mortality, *P. vivax* biology complicates malaria control and treatment because hypnozoites can remain in a latent, undetectable state for months to years in the liver. Hypnozoites can activate and seed a blood-stage infection, which can cause symptomatic disease (called a relapse) and onward parasite transmission. Thus, elimination of latent *P. vivax* from the liver is necessary to achieve the goal of malaria eradication. Indeed, relapse remains a significant barrier to eradication as the majority of *P. vivax* infections observed clinically are due to relapse.[1,2] Current drugs used to prevent relapse can cause hemolysis in persons with glucose-6-phosphate dehydrogenase deficiency,[3] an enzymatic deficiency prevalent in Southeast Asia and South America where *P. vivax* is prevalent,[4] limiting their widespread use. Therefore, the discovery of novel therapeutics that prevent *P. vivax* relapse and are well tolerated in populations living in endemic areas will be important for malaria eradication.[5,6]

The understanding that *P. vivax* could cause malaria again months after primary disease was established more than a century ago,[7] yet the latent hypnozoite form of the parasite was not observed in the liver until 1982,[8] when a splenectomized chimpanzee was experimentally infected with *P. vivax* sporozoites and liver biopsies were used to visualize parasites in the hepatic tissue. Although these small tissue forms had not been directly observed to activate and cause blood-stage infection, they were presumed to be the liver stage forms which can activate and seed blood-stage infections at later time points after the initial infection. A handful of experimental reports thereafter described the observation of *P. vivax* hypnozoites in an *in vitro* culture system.[9–11] Further developments in technology and persistence in improving the logistics of *in vitro* infection have led to *in vitro* platforms that are capable of medium-throughput screening to identify novel chemical matter that prevents relapse.[12–15] These platforms have also enabled the demonstration of full liver stage development

Received 24 March 2022; accepted 31 July 2022;
https://doi.org/10.1016/j.omtm.2022.07.016.

[7]Present address: Novartis Institutes for Biomedical Research, Novartis Institute for Tropical Diseases, Emeryville, CA 94608, USA

[8]Present address: Vaccine and Gene Therapy Institute, Oregon Health and Science University, Portland, OR 97006, USA

[9]Present address: Evotec SE, Essener Bogen 7, 22419 Hamburg, Germany

Correspondence: Erika L. Flannery, Center for Global Infectious Disease Research, Seattle Children's Research Institute, Seattle, WA 98109, USA.
E-mail: erika.flannery@novartis.com
Correspondence: Stefan H.I. Kappe, Department of Pediatrics, University of Washington, Seattle, WA 98105, USA.
E-mail: stefan.kappe@seattlechildrens.org
Correspondence: Jetsumon Sattabongkot, Mahidol Vivax Research Unit, Faculty of Tropical Medicine, Mahidol University, Bangkok, Thailand.
E-mail: jetsumon.pra@mahidol.ac.th

© 2022 The Authors.
This is an open access article under the CC BY license (http://creativecommons.org/licenses/by/4.0/).

of *P. vivax* and *P. cynomolgi* (a latent relapsing parasite species that infects monkeys) *in vitro* from sporozoite infection of hepatocytes to fully functional schizonts that can release red blood cell-infectious exo-erythrocytic merozoites into the culture medium.[13] Recently, using live imaging *in vitro*, Voorberg-van der Wel et al. showed hypnozoites present in primary cells can activate to form secondary replicating liver schizonts.[16] To further enable hypnozoite research, a liver-chimeric humanized mouse model was developed that supports *P. vivax* acute and latent liver infection (FRG huHep),[17] which leverages mice re-populated with human primary hepatocytes.[18,19] These recent developments have favorably impacted the feasibility of *P. vivax* liver stage research and drug discovery.[20]

Experimental studies with *P. vivax* are additionally complicated by the fact that unlike *P. falciparum*, *P. vivax* cannot be cultivated in the laboratory in human blood. The blood stage of the parasite is necessary for mosquito infection and subsequent development of the liver-infectious sporozoites in the mosquito salivary glands. Therefore, blood-stage parasites from human donors infected with *P. vivax* are obtained, fed as a blood meal to female Anopheline mosquitoes to induce an infection in the mosquito. In the mosquito, the sporozoites grow and migrate from the midgut to the salivary glands over a 14-day period. To study drug effects on the parasites, sporozoites are then harvested from the mosquito salivary glands by manual dissection and used to infect *in vitro* primary hepatocyte cultures or introduced intravenously or through the bite of a mosquito to the bloodstream or skin of FRG huHep mice.

Post sporozoite infection, *P. vivax* liver stages differentiate from uni-nucleate trophozoites resident in host hepatocytes to liver stage parasites of two distinct fates as reviewed in Schäfer et al.[21] A fraction of the parasites begin a rapid growth process called exo-erythrocytic schizogony, where they develop into a large schizont that occupies the entire hepatocyte and even stretches the hepatocyte membrane. At the end of this enormous cell growth and DNA replication, the parasite differentiates into tens of thousands of red blood cell-infectious exo-erythrocytic merozoites.[22,23] An alternative fate of the parasite is for the trophozoite to remain uni-nucleate and enter latency as a hypnozoite, which, until the last decade of technology development, remained exceedingly difficult to study. In the FRG huHep model, these two parasite states become distinct from one another as early as three days post-infection as their sizes and DNA content diverge dramatically.[17] Eight to ten days post-infection merozoites egress from mature primary schizonts into the bloodstream and infect red blood cells.[17] For the latent hypnozoite form, activation can then occur weeks, months, or years after primary infection, which leads to entry into rapid secondary schizogony and release of second-generation exo-erythrocytic merozoites. Triggers for this activation are unknown but numerous factors have been postulated to play a role.[24]

Observations of the phenotypic effects of drugs on hypnozoites have been limited to *in vitro* work. While many clinical studies have been conducted to understand the frequency of relapse and the effect drugs have on relapse prevention,[25–27] very little is understood about the mechanism of action of 8-aminoquinolines on the intrahepatic parasites. The *P. cynomolgi* monkey relapse model is the pre-clinical model for showing proof-of-concept of activity against hypnozoites before entry into the clinic.[28] In this model, similar to clinical studies, parasites cannot be routinely observed in the liver and blood-stage patency is used as a surrogate to assess the effect of drugs on hypnozoites in the liver.

Here, we use the FRG huHep mice to understand hypnozoite activation *in vivo*, within the host hepatocyte and provide a much-needed bridge from *in vitro* experiments to those conducted in monkeys and clinical studies in humans, which are based on blood-stage relapse observations. Our studies provide novel insight that will impact the study design of clinical trials as well as future *in vivo* work in monkeys and mice to discover novel compounds with radical cure activity against *P. vivax*.

RESULTS

P. vivax field isolates reproducibly establish primary liver-stage infection in a liver-chimeric humanized mouse model (FRG huHep)

We investigated the utility of an *in vivo* model of *P. vivax* infection that uses distinct parasite isolates for each infection. *P. vivax*-infected blood from donors in several regions in Thailand was obtained and fed to female *Anopheles dirus* mosquitoes to generate hepatocyte-infectious sporozoites. Mosquito salivary glands were dissected and *P. vivax* sporozoites were used to infect liver-chimeric humanized mice (FRG huHep)[29] to study *P. vivax* liver infection, including hypnozoites. Currently, no molecular marker differentiating latent, uni-nucleate hypnozoites from replicating, multi-nucleated schizonts is available. However, microscopic examination of infected liver tissue can easily differentiate hypnozoites and schizont-stage parasites by size, DNA content, and sub-cellular localization of UIS4 (upregulated in infectious sporozoites 4) protein.[17,30] To assess feasibility and to understand the relationship between the sporozoite dose and liver-stage parasite infection frequency in FRG huHep liver tissue sections, mice were infected intravenously with an increasing number of sporozoites from the same parasite isolate (VTTY116) and the primary liver-stage infection was quantified by microscopy 8 days post-infection (Figures 1A and 1B). A dose-dependent response was observed between sporozoite inoculum and number of parasites observed (hypnozoites and primary schizonts) in the liver tissue sections (hypnozoite range: 0.0–3.6 hypnozoites per 100 mm^2, Spearman r = 0.8; schizont: 0.9–39.2 schizonts per 100 mm^2, Spearman r = 0.964) (Figures 1A and 1B). Parasites were quantifiable at all doses, although the 50,000 sporozoite dose yielded very few observable parasites (1 hypnozoite in 600 mm^2 of liver tissue section visualized for one animal and no hypnozoites observed in 588 mm^2 in another). Although *Plasmodium* 18S qRT-PCR cannot detect the difference between schizont and hypnozoite rRNA, it allows quantification of overall liver-stage burden. Importantly, during the primary infection, nucleic acid from actively replicating schizonts is approximately 10,000 times more abundant than that from hypnozoites,[31] and thus most RNA measured in a primary infection are signals from schizonts. A dose-dependent relationship between 18S rRNA and sporozoite dose was

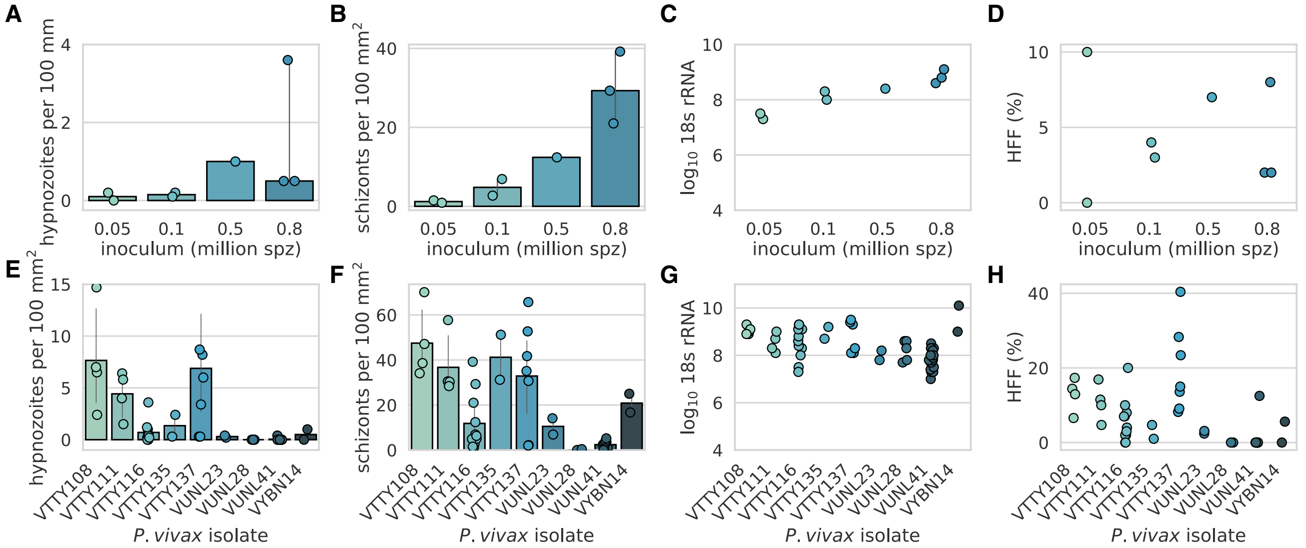

Figure 1. *Plasmodium vivax* field isolates demonstrate robust primary infection in liver-chimeric humanized mice

Number of (A) hypnozoites (Spearman correlation r = 0.8) and (B) schizonts (Spearman correlation r = 0.964) observed per 100 mm^2 of liver tissue section visualized 8 days post-infection using immunofluorescence assay (IFA). (C) Log_{10} *Plasmodium* 18S rRNA copies per μg of liver quantified after liver harvest and RNA extraction (Pearson correlation r = 0.872). (D) Hypnozoite formation frequency (HFF) (%) observed per inocula (p = 0.506, Kruskal-Wallis test). Number of (E) hypnozoites and (F) schizonts present during the acute infection (5–8 days post-infection) for nine distinct *P. vivax* field isolates. (G) Log_{10} *Plasmodium* 18S rRNA copies per μg of liver and (H) HFF for the same infections in (E–G). Each data point represents one animal. For microscopy counts, >3 liver tissue sections were counted per animal. The same parasite isolate (VTTY116) was used for (A–D). The number of sporozoites used for each infection in (E–H) was: VTTY108, VUNL23, VUNL28, VUNL41: 1,000,000; VTTY111: 600,000; VTTY116: 800,000; VTTY135; VTTY137: 500,000, and VYBN14: 850,000. Error bars represent 95% confidence interval.

observed (Figure 1C, Pearson r = 0.872, log_{10} 18S rRNA copies range: 7.3–9.1), like that observed between sporozoite dose and total number of schizonts observed, suggesting that 18S rRNA quantification correlates with the number of schizonts present. For the 50,000 sporozoite inoculum group, log_{10} 18S rRNA copies were 7.3 and 7.5. The hypnozoite formation frequency (HFF) (the percentage of parasites that are hypnozoites) was not significantly different between inocula (median =3.5%, Kruskal-Wallis test p = 0.506) (Figure 1D).

We next compared several additional experimental design variables to understand their utility in this small animal model of *P. vivax* infection. We observed no difference in liver parasite burden when animals were infected retroorbitally versus by intravenous tail vein injection (Figure S1A, p = 0.274, unpaired t test) as well as no effect of humanized mouse genetic background strain (NOD (FRGN) versus C57Bl/6 (FRG)) on infection levels (Figure S1B, p = 0.827, unpaired t test). To compare our findings with those observed in several other *in vivo* rodent models of malaria that infect with other *Plasmodium* spp., we used midgut sporozoites isolated from oocysts in the mosquito midgut to infect animals. Compared with that of salivary gland sporozoites (the stage of sporozoites that are naturally injected by the mosquito), the same number of midgut sporozoites injected resulted in a 2.5-log reduction in liver burden (p < 0.01, unpaired t test), similar to what has been observed for other *Plasmodium* species (Figure S1C).[32,33]

To understand the infection variability between distinct clinical isolates, we performed infection experiments with nine independent isolates. Each primary infection measured at 5–8 days post-infection resulted in an infection that was quantifiable by microscopy (Figures 1E and 1F) as well as 18S rRNA qRT-PCR (Figure 1G). The number of sporozoites used for each infection was: VTTY108, VUNL23, VUNL28, VUNL41: 1,000,000; VTTY111: 600,000; VTTY116: 800,000; VTTY135; VTTY137: 500,000, and VYBN14: 850,000. The median hypnozoite burden was 0.5 per 100 mm^2 (range: 0.0–21.3 per 100 mm^2) and the median schizont burden was 16.7 per 100 mm^2 (range: 0.0–70.1 per 100 mm^2). In some cases schizonts were not observed in the liver sections analyzed by microscopy, yet the parasite burden measured by 18S rRNA qRT-PCR remained robust (mean log_{10} 18S rRNA copies measured for the primary infection was 8.3 [range: 7.0–10.1], range of coefficient of variation [CV] for each isolate [1.9%–8.2%], overall CV = 8.0%). Although liver infection was observed with all isolates, the HFF range observed was high (0%–40.4%), exemplifying the natural level of variation in field isolates (Figure 1H), yet continued to show significant differences when grouped by circumsporozoite protein (CSP) allele type (p < 0.01, Mann-Whitney test) (Figure S1D).[17]

FRG huHep mice support latent *P. vivax* infection characterized by persistent hypnozoites and secondary schizonts

To assess the frequency and propensity for relapse, we characterized the latent stage of *P. vivax* liver infection through 49 days post-infection. We chose to use a large sporozoite dose (0.8–1.0 million sporozoites) to aide in phenotypic characterization of the parasites by microscopy. Latent hypnozoites were observed at all time points

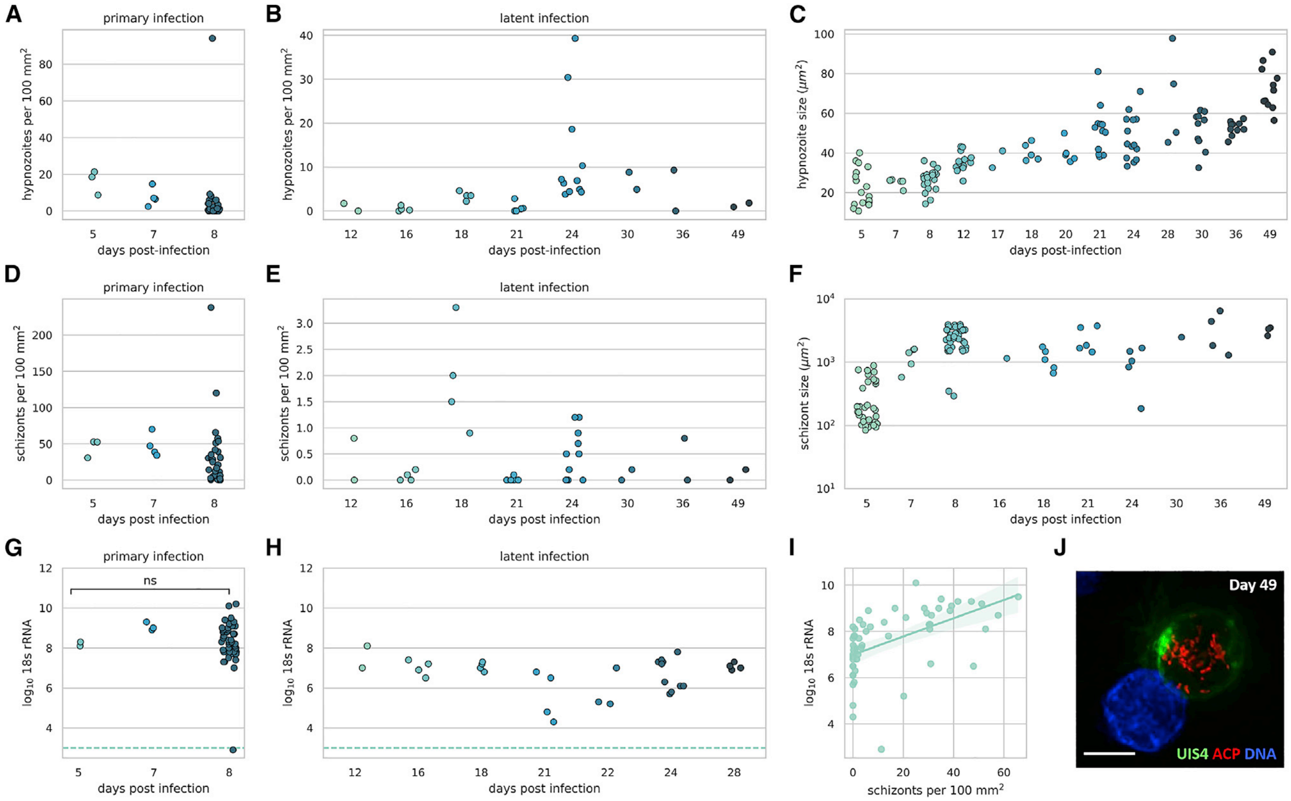

Figure 2. Latent *P. vivax* infections are characterized by persistent hypnozoites and secondary schizonts

Number of hypnozoites observed in liver tissue sections during the (A) primary infection and (B) latent infection (Mann-Whitney test p = 0.767 for significance between the number of hypnozoites present in the primary and latent infection). (C) Size of *P. vivax* hypnozoites observed over 7 weeks after infection with 0.8–1.0 million sporozoites. Number of schizonts observed in liver tissue sections during the (D) primary and (E) latent infection (Mann-Whitney test p < 0.001 for significance between the number of schizonts present in the primary and latent infection). (F) Size of *P. vivax* schizonts over 7 weeks after infection with 0.8–1.0 million sporozoites. Total $\log_{10}$ *Plasmodium* 18S rRNA per μg of RNA during the primary (G) and latent (H) infection (p < 0.0001, unpaired t test for significance between the total 18S rRNA measured in the primary and latent infection). (I) Correlation curve for the number of schizonts observed in liver tissue sections and the corresponding 18S rRNA levels for each animal (Pearson r = 0.53). Horizontal dashed line denotes the limit of qunatification. (J) Hypnozoite 49 days post-infection. Immunofluorescence staining for ACP (red), UIS4 (green), and DNA (DAPI, blue). Scale bar, 5 μm. For the latent infections the parasite isolates used were (VUNL23, VUNL28, VBTM3, VKTS102, VYBN70, VTTY52, VTTY111, VTTY137). Each symbol represents observations from one animal in all figures except (C and F). Each symbol represents the area of one parasite which was measured in (C and F).

sampled post-infection with eight different Thai *P. vivax* isolates (VUNL23, VUNL28, VBTM3, VKTS102, VYBN70, VTTY52, VTTY111, and VTTY137) used to complete the time course (Figure 2A primary infection, Figure 2B latent infection). The number of hypnozoites in the infected livers was not significantly different between the primary infection (day 8) (Figure 2A) and the latent infection (Figure 2B) (median = 1.0 hypnozoites per 100 mm^2 and 3.5 hypnozoites per 100 mm^2, respectively, p = 0.767, Mann-Whitney test). Hypnozoites modestly and linearly increased in cell size from day 5 through day 49 (Figure 2C), suggesting metabolic activity and development. This growth, however, was unlike the exponential growth of exo-erythrocytic schizonts observed within the first week of infection (Figure 2F). Visualization of hypnozoites 49 days post-infection in the mouse liver using antibodies to the parasitophorous vacuole membrane protein UIS4 and apicoplast (ACP) proteins revealed parasites with extensive apicoplast network development (Figure 2J) compared with hypnozoites visualized 8 days post-infection.[17]

The primary wave of schizonts release exoerythrocytic merozoites into the blood 8–10 days post-infection.[17] Therefore any schizonts observed during the latent phase of infection (>10 days post-infection) are presumably secondary schizonts derived from activated hypnozoites. The triggers and the degree of synchronicity of hypnozoite activation remain unknown.[24] Therefore, any single infected liver section analyzed during the latent stage of infection can contain secondary schizonts originating from a subpopulation of activated hypnozoites. To assess this experimentally, we performed infections with eight different *P. vivax* isolates and harvested livers from days 12 to 49 post-infection to understand the dynamics of hypnozoite activation. At every time point sampled, secondary schizonts were observed in at least one infected mouse from each isolate (median = 2.4, range = 0.0–237.9 per 100 mm^2) (Figure 2D primary infection, Figure 2E latent infection). The number of secondary schizonts present at latent time points was significantly less than the number of primary schizonts present at day 8 of a primary infection (median = 0.15 per

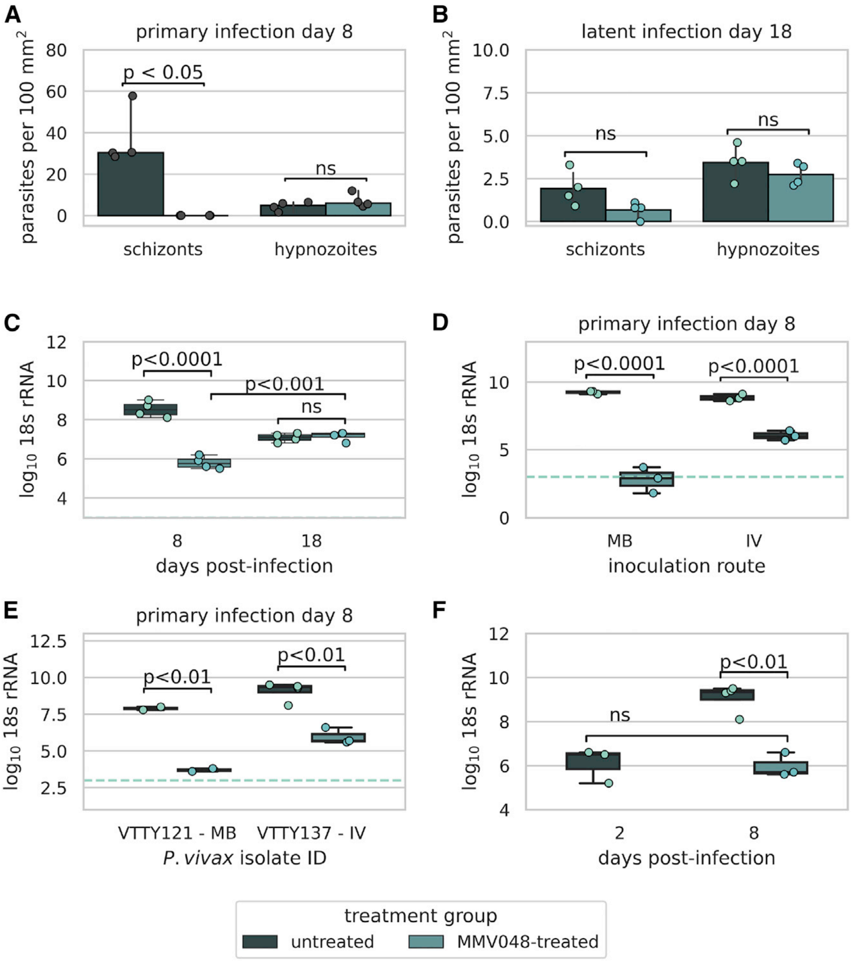

Figure 3. Functional characterization of *P. vivax* hypnozoite activation *in vivo*

(A) Quantification of hypnozoites and schizonts 8 days post-infection (primary infection) (B) and 18 days post-infection (latent infection) in FRG huHep livers with and without MMV048 treatment (isolate VTTY111). (C) Log_{10} 18S rRNA per μg of liver 8 and 18 days post-infection from (A and B). (D) Log_{10} 18S rRNA per μg of liver 8 days post-infection after inoculation with 800,000 VTTY116 sporozoites intravenously (IV) or by the bites of 20 mosquitoes (MB) with and without MMV048 treatment. (E) Log_{10} 18S rRNA per μg of RNA in two different *P. vivax* isolates 8 days after infection and with and without MMV048 treatment. (F) Log_{10} 18S rRNA per μg of RNA 2 days post-infection and 8 days post-infection with and without MMV048 treatment (isolate VTTY137). Each dot represents measurements from one animal. Treatment with MMV048 was with 30 mg/kg on days 4–7 post-infection. Horizontal dashed line denotes the limit of quantification. Barplot error bars represent 95% confidence interval.

100 mm² and 16.7 per 100 mm², respectively, $p < 0.001$, Mann-Whitney test). Primary schizonts, unlike hypnozoites, increase exponentially in size during the primary infection (as observed in Mikolajczak et al.[17]) and this size increase can be observed for secondary schizonts during the latent infection (Figure 2F). Measurement by total *Plasmodium* 18S rRNA in the liver also showed a significant decrease in the amount of 18S rRNA copies present during the latent infection compared with day 8 post-infection (mean = 6.5 versus 8.3 log_{10} 18S rRNA copies, respectively, $p < 0.0001$, unpaired t test) (Figures 2G and 2H). In addition, the amount of 18S rRNA copies correlated with the number of schizonts observed in each animal (Figure 2I) (Pearson r = 0.53).

Validation of hypnozoite-derived secondary schizont formation

To demonstrate that the secondary schizonts we observed at later time points post-infection were indeed derived from activated hypnozoites and not slow-growing or growth-arrested primary schizonts, we used treatment with MMV390048 (MMV048), a compound that targets phosphatidylinositol 4-kinase in the parasite.[34,35] Compounds with this mechanism of action have activity against *P. vivax* schizonts and hypnozoites when used prophylactically; however, when used after the establishment of liver infection, these compounds demonstrate preserved schizonticidal activity with minimal effect on established hypnozoites. This renders them an ideal tool for the study of hypnozoite activation and relapse.[12,13,36] At 8 days post-infection, treatment with MMV048 (30 mg/kg) on days 4–7 days post-infection resulted in a significant reduction in the number of primary schizonts (no schizonts were observed in the liver sections of four animals examined) compared with the control group (median 0 schizonts and 30.4 schizonts per 100 mm², respectively [$p < 0.05$, Mann-Whitney test]) (Figure 3A). Elimination of primary schizonts from the liver with MMV048 did not have a significant effect on the number of hypnozoites observed during the primary infection (median = 6.0 per 100 mm² compared with 4.9 per 100 mm² in the untreated group, p = 0.3429 Mann-Whitney) (Figure 3A). When measured by 18S rRNA, there was a 99.81% reduction (2.7 log-reduction) in parasite burden in the MMV048-treated group compared with the untreated animals during the primary infection ($p < 0.0001$, ANOVA Tukey's correction for multiple tests, mean 5.8 versus 8.5 log_{10} 18S rRNA, respectively) (Figure 3C).

In a second group of mice that was subject to the same treatment regimen and with the same parasite isolate (VTTY111) used for infection, liver harvest and examination during the latent infection (18 days post-infection) revealed detectable multi-nucleated secondary schizonts in the MMV048-treated group at a similar frequency as the control group (0.80 and 1.75 schizonts per 100 mm², respectively, p = 0.0571, Mann-Whitney test), demonstrating the generation of secondary schizonts from activated hypnozoites (Figure 3B). At day 18, parasite 18S rRNA levels in untreated animals had declined, as expected, compared with day 8 (due to the egress of primary schizonts) (mean 8.5 versus 7.1 log_{10} 18S rRNA [$p < 0.001$, unpaired t test], a

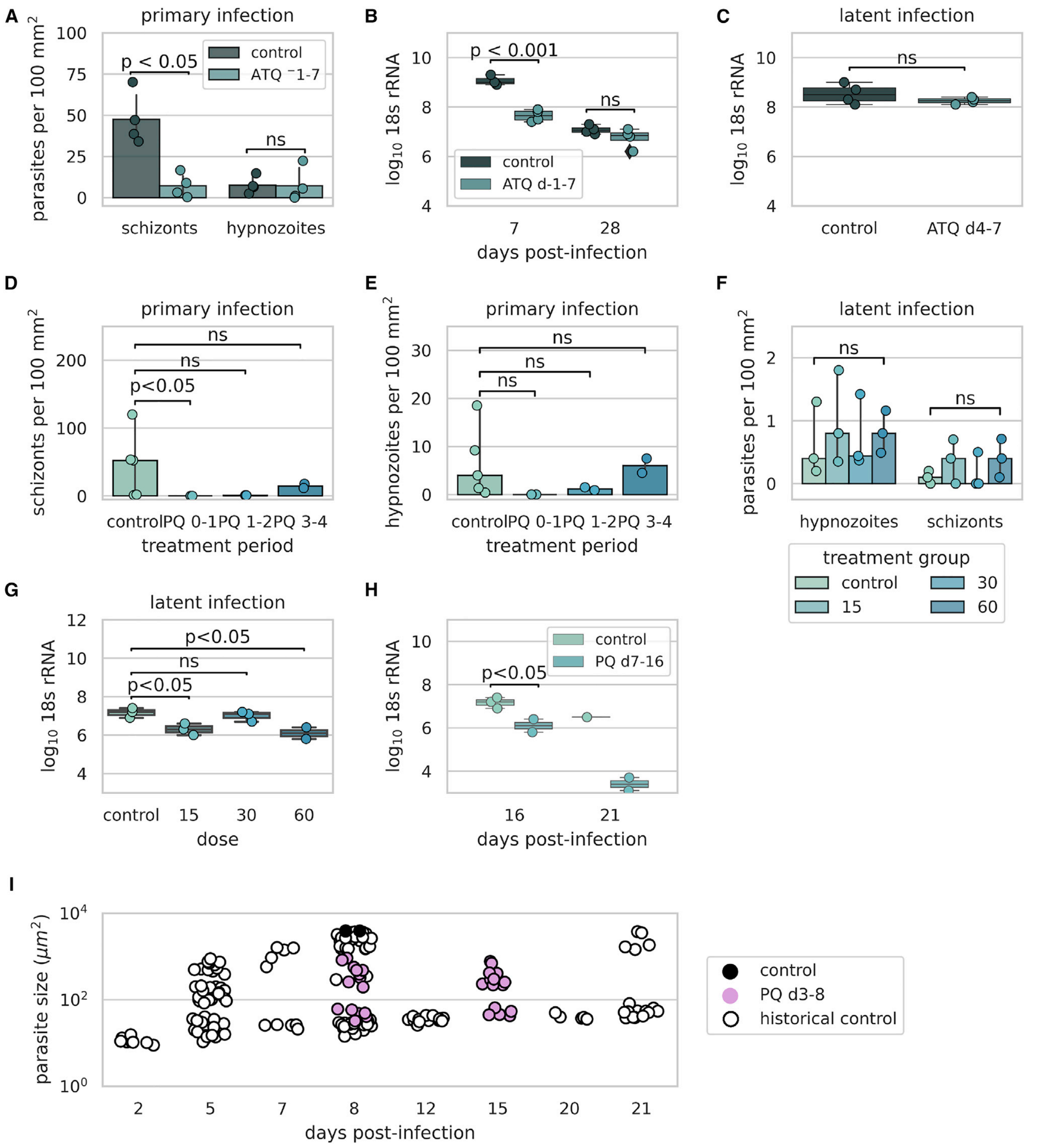

Figure 4. Effect of primaquine and atovaquone on liver-stage parasite phenotype

(A) Number of parasites present with and without 10 mg/kg atovaquone (ATQ) treatment (days −1 to 7). Control versus treatment for schizonts ($p < 0.05$, Mann-Whitney) and control versus treatment for hypnozoites ($p = 0.468$, Mann-Whitney). (B) Log$_{10}$ 18S rRNA per µg of liver 7 days post-treatment with 10 mg/kg ATQ from days −1 to 7 (control versus ATQ treated, $p < 0.001$, ANOVA, Tukey's multiple comparisons) and 28 days post-treatment (control versus ATQ treated, $p = 0.325$, ANOVA, Tukey's multiple comparisons). (C) Log$_{10}$ 18S rRNA per µg of liver 7 days post-infection following treatment with or without 10 mg/kg ATQ from days 4 to 7 (control versus treatment, $p = 0.2415$, unpaired t test). (D) Number of schizonts and (E) hypnozoites present in the liver 8 days post-infection (primary model) after 2-day treatment with primaquine (PQ) (60 mg/kg)

(legend continued on next page)

96.45% reduction in rRNA); however, animals previously treated with MMV048 demonstrated a 1.3 log increase in parasite 18S rRNA compared with treated animals on day 8, a finding that confirms the microscopic observations of emergence of secondary schizonts. This further confirmed that hypnozoite activation generated secondary schizonts (mean 5.8 [day 8] versus 7.2 [day 18] log_{10} 18S rRNA [$p < 0.001$, unpaired t test]) (Figure 3C) in the treated group.

We saw a similar reduction in the amount of log_{10} 18S rRNA remaining during the primary infection when treating with MMV048 using a different Thai *P. vivax* isolate (VTTY116); a 99.84% reduction (mean 8.8 and 6.0 log_{10} 18S rRNA for control and treated animals, respectively) (Figure 3D). When infecting FRG mice via mosquito bite with the same isolate, the reduction in log_{10} 18S rRNA was even greater following MMV048 treatment (100.00% reduction, mean 9.2 versus 2.8 in control and treated animals, respectively) (Figure 3D). Further confirmation with two additional isolates, VTTY121 and VTTY137, reproduced the significant levels of reduction observed with mosquito bite-delivered infection (VTTY121, 99.99% reduction, 4.2 log-reduction, $p < 0.01$, unpaired t test) and intravenous infection (VTTY137, 99.92% reduction, 3.1 log-reduction, $p < 0.01$, unpaired t test), respectively (Figure 3E).

To further validate that the amount of 18S rRNA measured in the MMV048-treated mice at day 8 is derived from hypnozoites only, we compared the level of 18S rRNA present in the MMV048-treated animals to the levels of 18S rRNA observed in untreated mice on day 2 post-infection. At this time point parasites are similar in size to latent hypnozoites and show similar amounts of DNA by immunofluorescent staining because they have not begun to replicate. Log_{10} 18S rRNA levels were comparable in the day 8 MMV048-treated mice and the day 2 untreated mice (6.1 versus 6.0 log_{10} 18S rRNA, respectively, $p = 0.968$, unpaired t test) (Figure 3F), further supporting the conclusion that all schizonts are cleared by MMV048 treatment and the amount of 18S rRNA present is derived from uni-nucleate hypnozoites. We conclude that the occurrence of hypnozoite-derived secondary schizonts more than 10 days after sporozoite infection constitutes a tissue stage proxy for hypnozoite activation that could result in blood-stage relapse as schizonts generate infectious exoerythrocytic merozoites that initiate blood-stage infection.

Effect of atovaquone and primaquine on liver-stage parasite phenotypes

Current standard of care treatment for prevention of *P. vivax* primary blood-stage infection and prevention of relapse due to latent infection are different. While atovaquone (ATQ)-proguanil is used prophylactically to prevent an acute onset of blood-stage infection, primaquine and tafenoquine are used to prevent relapses by treating a person who has patent *P. vivax* blood-stage parasitemia because it can be assumed that such individuals also carry latent hypnozoites in the liver. The efficacy of prophylactic and relapse-prevention drugs are measured clinically by the absence of patent blood-stage parasitemia as the human liver is not accessible to determine liver-stage burden. Thus, little is known about the phenotypic effect of these compounds on liver-stage parasites and how they prevent onset of blood-stage infection.

Therefore, we conducted several studies to understand the phenotypic effect of standard of care treatments on *P. vivax* parasites in the liver (Figure S2). *In vitro* studies have shown that the day of dosing has a significant effect on efficacy of liver-stage drugs,[12,13] likely because hypnozoites do not become latent until a few days after hepatocyte infection and their susceptibility to drugs differs because of this change in physiology.[37] Therefore, we used two methods of dosing, prophylactic, which begins before or immediately after sporozoite infection, and radical cure, which begins when hypnozoites are mature and are less sensitive to liver-stage antimalarial drugs. First, we used ATQ, a prophylactic drug that prevents primary infection. Treatment with 10 mg/kg ATQ began 1 day before infection (day −1) and continued until 7 days post-infection and liver tissue sections were evaluated 7 days post-infection. Under these conditions we observed a decrease in the number of schizonts present in the ATQ-treated group compared with the untreated group (median = 6.0 and 42.3 per 100 mm^2, respectively, $p < 0.05$, Mann-Whitney test) with no decrease in the number of hypnozoites (median = 3.25 and 6.75 per 100 mm^2, respectively, $p = 0.486$, Mann-Whitney test) (Figure 4A). The decrease in the number of schizonts present after ATQ treatment was further ascertained by a 96.17% (1.4-log) decrease in overall *Plasmodium* 18S rRNA liver burden between the ATQ-treated and control groups (mean = 7.7 and 9.1 log_{10} 18S rRNA, respectively, $p < 0.001$, ANOVA, Tukey's multiple comparisons) (Figure 4B). Furthermore, an additional 21 days of incubation without drug treatment showed no difference in the overall levels of parasite 18S rRNA between the ATQ-treated and control groups (mean = 6.8 and 7.1 log_{10} 18S rRNA, respectively, $p = 0.325$, ANOVA, Tukey's multiple comparisons) (Figure 4B), suggesting that only hypnozoites are still remaining in the ATQ-treated group. Starting treatment 4 days post-infection and continuing until 7 days post-infection had no overall effect on total liver 18S rRNA (unpaired t test, $p = 0.2415$) (Figure 4C) and of the two animals where liver sections were

beginning at different days post-treatment. For treatment days 0–1, the number of schizonts in control versus untreated ($p < 0.05$, Kruskal-Wallis test), treatment days 1–2 ($p = 0.267$, Kruskal-Wallis test), and treatment days 3–4 ($p > 1.0$, Kruskal-Wallis test). For the number of hypnozoites in control versus untreated animals treatment days 0–1 ($p = 0.119$, Kruskal-Wallis test) and treatment days 1–2 and 3–4 ($p > 1.0$, Kruskal-Wallis test). (F) Number of parasites present 16 days post-infection after treatment with different doses of PQ from day 7 to 16. Control versus treated (all concentrations) schizonts ($p = 0.533$, Kruskal-Wallis test) and hypnozoites ($p = 0.867$, Kruskal-Wallis test). (G) Total log_{10} 18S rRNA per μg of liver following treatment with different concentrations of PQ from days 7 to 16 post-infection. Control versus 15 mg/kg ($p = 0.023$, ANOVA), 30 mg/kg ($p = 0.841$, ANOVA), and 60 mg/kg ($p = 0.0148$, ANOVA) (H) Log_{10} 18S rRNA per μg of liver 16 (control versus treated, $p = 0.0354$, unpaired t test) and 21 days post-infection after treatment with different doses of PQ from day 7 to 16. (I) Parasite sizes before and after treatment with PQ (60 mg/kg) for 5 days (3–8 days post-infection) measured 8 and 15 days post-treatment. The parasite isolate used for the control and PQ treament groups was VUBR6. Each dot represents measurements from one animal except in (I). Each dot represents the area measured for one parasite in (I). Barplot error bars represent 95% confidence interval.

evaluated, both animals had hypnozoites and schizonts in their liver, demonstrating that the drug is effective against schizonts only when given prophylactically and not active against hypnozoites.

We next examined the effect of drug regimens on parasites in the liver using primaquine. To ensure that primaquine reproduced results obtained previously in human volunteers[38,39] and for comparison with effects observed with *P. falciparum* in the FRG huHep model,[40] we used treatment with 60 mg/kg primaquine, beginning at different days during the primary infection, to observe the effect of dosing day. Treatment from days 0 to 1 resulted in a complete elimination of schizonts from the liver tissue sections sampled (median = 52.4 schizonts per mm^2 in the control, $p < 0.05$, Kruskal-Wallis test for schizonts in treated versus control) (Figure 4D), whereas beginning dosing at a later time point (days 1–2 or 3–4) did not result in the elimination of schizonts in tissue sections sampled (median = 0.9 schizonts per mm^2 days 1–2 [$p = 0.267$, Kruskal-Wallis test], median = 14.7 schizonts per mm^2 days 3–4 [$p > 1.0$, Kruskal-Wallis test]). Treatment from days 0 to 1 also resulted in a complete absence of hypnozoites present in liver tissue sections sampled (median = 4.0 hypnozoites per mm^2 in the control, $p = 0.119$, Kruskal-Wallis test for hypnozoites in the treated versus control). The number of hypnozoites present continued to increase as the starting time of dosing extended from the time of infection (median = 1.2 and median = 6.0 hypnozoites per mm^2 after dosing days 1–2 and 3–4, respectively) (Figure 4E). Although there was no significant decrease in the number of schizonts observed when treatment began on day 3 (median = 14.7 and 45.8 [control] per mm^2, $p > 0.990$, Kruskal-Wallis test), examination of schizonts treated from days 3 to 4 post-infection with 60 mg/kg primaquine showed developmentally arrested parasites that were smaller in size with disrupted apicoplast branching and ER networks (Figure S3).

When primaquine is used clinically, patients are at least 11 days post-infection, as this is when patients present with symptoms.[41–43] Therefore, we began dosing at day 7 to observe the effect of primaquine on established hypnozoites (a time point after the parasite has established latency yet earlier than clinically to reduce the total experimental period). No dose-dependent clearance effect on hypnozoites or schizonts by primaquine was observed when treating from days 7 to 16 and examining livers 16 days post-infection (Figure 4F). Liver parasite burden levels measured by 18S rRNA showed a significant decrease in the 15 and 60 mg/kg primaquine-treated groups (control median = 7.2 versus 6.3 log_{10} 18S rRNA for the 15 mg/kg group [$p = 0.0234$, ANOVA] and median = 6.1 log_{10} 18S rRNA for the 60 mg/kg group [$p = 0.0148$, ANOVA]) (Figure 4G). In addition, incubation for an additional 5 days resulted in a 99.92% (3.1 log) decrease in the overall liver-stage parasite burden in the 60 mg/kg treatment group, suggesting that all parasites affected by primaquine treatment may not be cleared from the liver for several days (Figure 4H). Because schizonts treated for 2 days with primaquine exhibited cellular stress, we again treated them with primaquine (from days 3 to 8) and examined the fate of stressed parasites at later time points. Schizonts were smaller in the primaquine-treated group compared with the untreated group on day 8 (mean = 492.2 and 2,514 μm^2, respectively [$p < 0.0001$, ANOVA]), and the primaquine-treated schizonts were similar in size to control schizonts 5 days post-infection (mean = 492.2 and 292.1 μm^2, respectively [$p = 0.8441$, ANOVA]) (Figure 4I). Further incubation to day 15 did not result in re-growth or clearance of the parasites as primaquine-treated schizonts were still present and were not different in size from those observed at day 8 (mean = 380.5 and 492.2 μm^2, respectively [$p = 0.9818$, ANOVA]) (Figure 4I).

FRG huHep *P. vivax* infection model for radical cure

Based on our learnings from previous experiments, we designed a model that did not depend on immediate hypnozoite clearance as a readout and was not confounded by primaquine-arrested schizonts to understand the effect of antimalarials on hypnozoite activation. We used the schizont-selective compound MMV048 to establish a 24-day tissue proxy for a hypnozoite activation model. Animals were treated with chloroquine (CQ), primaquine (PQ), or MMV048, or in combination at 10, 30, and 30 mg/kg, respectively (Figure S4). Treatment with primaquine began 4 days post-infection, a time point as early as possible, yet ensuring fully established hypnozoites in the liver, and given for 14 days to mimic the treatment regimen in the clinic. Treatment with chloroquine was from 4 to 6 days post-infection, similar to the 3-day treatment administered in combination with primaquine in patients with *P. vivax* infection. Treatment with MMV048 was given from 4 to 7 days post-infection to remove primary infection schizonts that would be arrested by primaquine (as observed in Figure 4).

These studies were conducted using several different Thai isolates. Due to limitations in the total numbers of FRG mice that can be used in a single study, we ensured that the number of mice per treatment group was large to have good reproducibility and spread treatment groups across studies while always having an untreated control group in each experiment. We normalized all liver 18S rRNA readouts to the untreated controls in each experiment (for each isolate) and calculated both the percent reduction and the log-reduction for each treatment group. Statistics were calculated using the log-transformed values. Treatment with chloroquine alone had no effect on parasite 18S rRNA levels in the liver, consistent with chloroquine's blood-stage-restricted activity observed clinically.[44] Treatment with primaquine alone or primaquine in combination with chloroquine reduced parasite 18S rRNA levels by 95.0% (1.4 log, $p < 0.05$, ANOVA) and 97.13% (1.9 log, $p < 0.0001$, ANOVA), respectively, consistent with observations that primaquine arrests schizonts and they are maintained in the liver. Treatment with MMV048 alone also resulted in a reduction in the parasite burden (87.0%, 1.2 log, compared with untreated controls, $p < 0.0001$, ANOVA). However, the greatest reduction in liver parasite burden was when animals were treated with primaquine in combination with MMV048 or primaquine in combination with MMV048 and chloroquine, which resulted in a near-complete elimination of parasites from the liver (99.99% [$p < 0.0001$, ANOVA] and 99.98% [$p < 0.0001$, ANOVA] reduction, respectively, compared with untreated mice), a finding

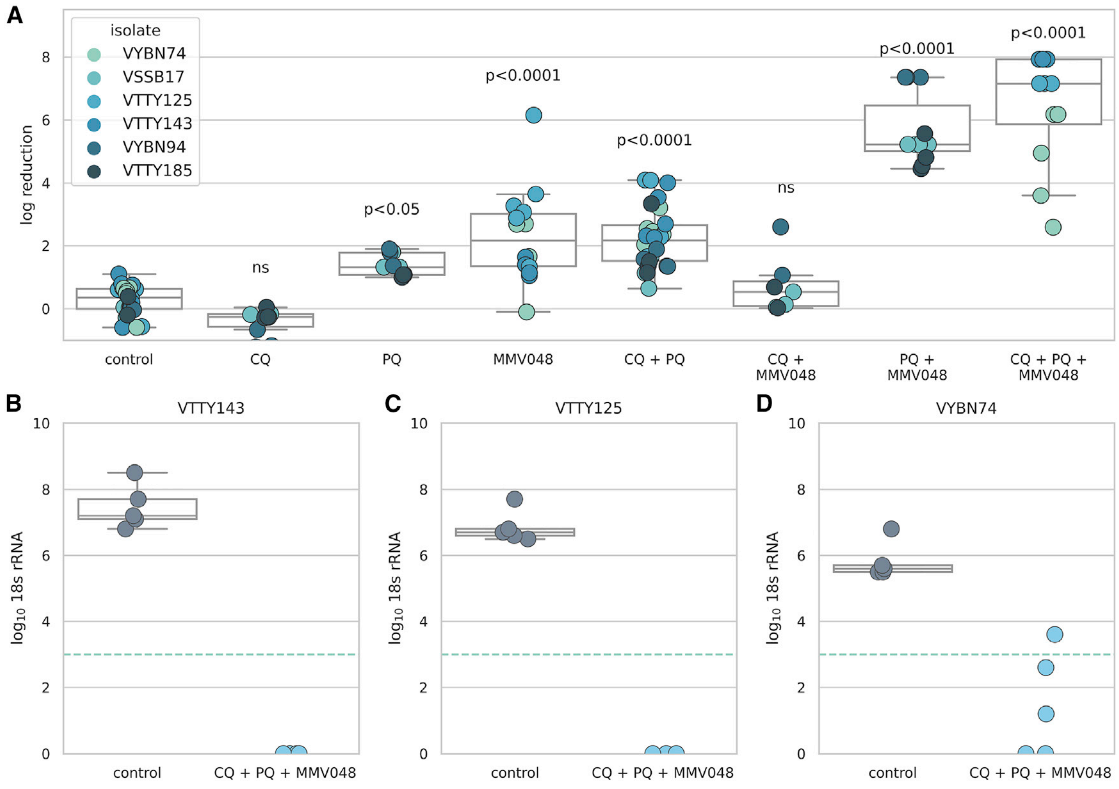

Figure 5. Treatment with primaquine, chloroquine, or MMV048 alone or in combination

(A) Log reduction in liver parasite burden per animal 24 days post-infection. Values are normalized to the controls of the same isolate from the same infection. Drug treatment was as follows: MMV048: 30 mg/kg days 4–7; chloroquine (CQ): 10 mg/kg days 4–6; primaquine (PQ): 30 mg/kg days 4–18. The box and whiskers plot denotes the mean, Q1 and Q3 of the values pooled for each treatment group. The number of animals in each group were as follows: {control:24, PQ:9, CQ:10, MMV048:14, PQ + MMV048:11, CQ + MMV048: 7, CQ + PQ: 26, CQ + PQ + MMV048: 12}. Horizontal dashed line denotes the limit of quantification. Log_{10} 18S rRNA per μg of liver of animals in the control group and the triple combination group for isolates (B) VTTY143, (C) VTTY125, and (D) VYBN74. Each dot represents one animal.

consistent with radical cure activity (Figure 5A). Of the three isolates tested in the triple-therapy group, liver-stage burden was eliminated below the limit of detection for two of the Thai isolates (Figures 5B and 5C), while the third isolate showed a 99.94% (mean = 4.7 log) reduction in liver 18S rRNA burden (Figure 5D).

DISCUSSION

Here, we describe a small animal model of *P. vivax* liver-stage infection, which can utilize field isolates to reproducibly model *P. vivax* infection, hypnozoite latency and activation. Latent infection is characterized by hypnozoite persistence and linear cell growth as well as activation of hypnozoites and development into secondary schizonts at all time points observed during the latent infection. A major challenge in detecting relapse in an experimental system that quantifies parasites in the liver is the potential for confounding by residual schizonts that are growth arrested during the primary infection or after drug treatment. We were able to overcome this challenge using the schizont-selective drug MMV048 to eliminate schizonts originating from the primary infection as well as those that were arrested due to primaquine treatment and were thus able to identify and quantify secondary schizonts originating from hypnozoites. Measuring this allowed us to demonstrate the time-dependent hypnozoiticidal efficacy of primaquine and thus validate a model for *in vivo* pre-clinical testing.

While parasites can be observed in the tissue microscopically at all time points sampled, we show that measurement of liver parasite burden using qRT-PCR is a more robust measure of parasite burden in liver. *P. vivax* infection in hepatocytes is rare in *in vitro* culture,[12] similar to that predicted in humans[45–47] and similar to rates we observe in the FRG huHep mouse model. Liver microscopy visualization is a less-robust measure of infection due to the inherent nature of count data and the fact a very small fraction of the liver is processed and observed (>3 slices of 50 μM tissue sections). Conversely, half of each hepatic lobe is used for 18S rRNA analysis, significantly increasing the sampling area of the liver. Despite the lack of molecular markers to distinguish hypnozoite versus schizont liver burden, herein we show that a qRT-PCR approach for measuring hypnozoite burden is feasible by using MMV048 to remove schizonts, which are the predominant RNA signal due to their exponential cell growth and therefore size. In these studies, we initially continued to use microscopy to differentiate hypnozoites and schizonts so we could

Table 1. Descriptive statistics of animals treated with and without MMV048 days 4–7 post-infection

Infection route	Intravenous	Intravenous	Mosquito bite	Mosquito bite
Treatment	MMV048	untreated	MMV048	untreated
No. of values	10	13	5	6
Minimum	5.500	8.100	1.800	7.800
Maximum	6.600	9.500	3.800	9.300
Range	1.100	1.400	2.000	1.500
Mean	5.920	9.000	3.160	8.600
SD	0.3736	0.4655	0.8385	0.7043
SEM	0.1181	0.1291	0.3750	0.2875

All animals were assessed for 18S rRNA levels by qRT-PCR on day 8.

characterize the cellular phenotypes of these parasites. One measure determined by microscopy, the HFF, was lower than previously reported for *in vitro* cultures.[12,13] It could be that parasites are more likely to become hypnozoites *in vitro* due to sub-standard growth conditions for the hepatocytes leading to stress in the parasite,[48] a difference in parasite clearance rates by the host system *in vitro* versus *in vivo*, or another not understood mechanism.

Using MMV048 as an experimental compound to clear schizonts from the liver allowed us to approximate the magnitude of rRNA signal expected from a hypnozoite population. In all intravenous infections where MMV048 was used from days 4 to 7 post-infection, the range of rRNA measured at day 8 during the primary infection was from 5.5–6.6 log_{10} 18S rRNA (mean = 5.9, SD = 0.37). The range for untreated control animals was 8.1–9.5 log_{10} 18S rRNA (mean = 9.0, SD = 0.47), suggesting that the amount of 18S rRNA measured can distinguish between the presence and absence of schizonts. In experiments where mosquito bite was used for infection, and presumably the sporozoite inoculum is lower than intravenous injection, the range and difference observed between treated and untreated FRG mice was even more distinct. The range of 18S rRNA measured in MMV048-treated mice infected via mosquito bite was from 1.8 to 3.8 log_{10} 18S rRNA (mean = 3.2 log_{10} 18S rRNA), while the range in untreated mice was from 7.8 to 9.3 log_{10} 18S rRNA (mean = 8.6 log_{10} 18S rRNA). The mean difference between treated and untreated mice was 3.1 log_{10} 18S rRNA for mice infected intravenously and 5.4 log_{10} 18S rRNA for mice infected via mosquito bite (Table 1).

A limitation of this model is the large dose of sporozoites used to induce an infection. The propensity to relapse with *P. vivax* is dependent on the hypnozoite density in the liver, which also impacts the number of days until a relapse is observed.[47,49,50] Most clinical trials are conducted with patients living in endemic regions and therefore infected naturally by the bite of a mosquito, where only tens to hundreds of sporozoites are injected into the skin and migrate to the liver.[32,33] Not all sporozoites will initiate a productive infection because attrition occurs at many steps along the infection process, including in the skin, in the blood stream, during hepatocyte entry, during parasite development, and during parasite egress. Mathematical modeling has shown that a person will only have 1–10 hypnozoites present in their liver after infection.[47] In the FRG huHEP model we use 0.5–1 million sporozoites (to aid in visualization of liver stages by microscopy), likely increasing the relapse rate observed. We required the use of this large inoculum to further characterize the latent infection as well as liver-stage phenotype using tissue microscopy. With the establishment of a clear relationship between relapse and parasite 18S rRNA quantity in the liver, observations of parasites by microscopic analysis of liver tissue may no longer be required to assess drug efficacy. Further studies that more accurately represent the levels of hypnozoite burden in the field will be important for the incorporation of the FRG huHep model into the drug discovery pipeline.

When treating with clinically used drugs against *P. vivax*, our experimental system demonstrated prophylactic but not anti-hypnozoiticidal activity of ATQ, consistent with its known prophylactic clinical efficacy and lack of clinical radical cure activity, as well as with *in vitro* observations.[12,51] Our results provide insights into biological effects underlying primaquine's radical cure activity. While the mechanism of action of primaquine has been proposed,[52] these are the first observations of the phenotypic effect of primaquine on *P. vivax* liver stages *in vivo*. In our experiments, primaquine treatment did not result in the immediate clearance of hypnozoites or schizont forms from the liver. These findings may seem unexpected; however, there is no *in vivo* evidence that primaquine kills and clears *P. vivax* from hepatocytes immediately after treatment. Indeed previous reports of primaquine treatment in rodent malaria *in vivo* show arrested schizonts, although this drug treatment cannot be tested in "radical cure mode" with rodent malaria species due to a shorter liver phase cycle of 2 days compared with the 8–11 days before egress of *P. vivax in vivo*.[53] Observations following single-dose primaquine treatment of *P. falciparum* gametocytes in patients revealed that gametocytes are still present in the blood 24 h following primaquine treatment, yet these gametocytes are not capable of inducing a mosquito infection. Thus primaquine's single-dose efficacy for preventing *P. falciparum* transmission cannot be assessed by measuring immediate gametocyte clearance after treatment.[54]

A cytostatic, likely nonviable, form resulting from effect of primaquine treatment on *P. vivax* schizonts was confirmed by a 7-day extended incubation post-primaquine exposure. These treated schizonts retained a similar cell size but did not resume growth once treatment was discontinued. Furthermore, the arrested schizonts are an important contribution to confounding the 18S rRNA measurements in mice treated with primaquine alone. Under these conditions schizonts would continue to contribute a large 18S rRNA signal; nonetheless, they are likely not able to replicate and egress. Taken together, these findings suggest that primaquine's mechanism of action against *P. vivax* liver forms may be more complex than the traditional dogma of immediate parasite killing. Instead, it may work by irreversibly inhibiting the biological processes needed for activation or the subsequent processes of schizogony and cytokinesis by the parasite. An alternative explanation and limitation of the model is the lack of a functional immune system in the FRG

huHep mice. The immune response may aid in parasite clearance upon drug exposure and thus the lack of immune effectors could contribute to the static effect we observe for primaquine in this model.

Clearance of primaquine-arrested schizonts by MMV048 allowed for measurement of parasite 18S rRNA levels that were not confounded by drug-arrested schizonts. Treatment with primaquine alone only moderately reduced the liver-stage 18S rRNA burden due to the signal from primaquine-arrested schizonts, yet treatment with MMV048, chloroquine, and primaquine in combination resulted in a complete elimination of parasite burden from the liver in all mice in two out of three of the Thai isolates tested. In the parasite isolate VYBN74, where 18S rRNA signal was still present, the signal in two of the mice was reduced to undetectable levels, while in three of the animals low levels of 18S rRNA were present. The remaining low signal detected could be due to several reasons. These levels could be hypnozoite specific, as even a single schizont would result in levels of at least 4 $\log_{10}$ 18S rRNA. This hypnozoite-specific population may be unable to activate, which would be concurrent with primaquine's anti-relapse activity measured clinically (and the arrest of schizonts observed *in vivo*). The low levels could alternatively be a result of growth-arrested or degenerating schizonts that either originate from the primary infection or hypnozoite-derived secondary schizonts that are degenerating or significantly smaller than normal schizonts. Although we have previously shown the FRG huHep mice to metabolize primaquine normally,[40] it is also possible that the hepatocyte lot used to re-populate the mice for the infection with VYBN74 did not metabolize primaquine to the fullest degree. Weak metabolizers of primaquine have been identified and this aberrant metabolism has been shown to lead to relapses.[55] It has also been demonstrated in clinical studies that primaquine is not 100% effective, which could be due to liver metabolism or parasite sensitivity among other reasons.[56] Finally, the failure to clear all parasite 18S rRNA from the liver could be due to the sporozoite dose used. In clinical trials of *P. vivax* relapse prevention, subjects are either those who have acquired *P.* vivax infection naturally[57] or experimentally by the bites of five mosquitoes,[58] and thus have far fewer hypnozoites in their livers.[47] Because observation of relapse in clinical trials is dependent on the length of follow-up of the study and the hypnozoite reservoir in the liver, it is feasible that, under similar inoculum conditions in clinical trials (0.5–1.0 million sporozoite injected intravenously), primaquine would not completely prevent relapse.

To validate the FRG huHep model, several dosing groups were used to best characterize the model and the response to different dosing combinations. Because CQ is used in combination with primaquine clinically, we included a triple-combination group with MMV048 to assess anti-activation activity. Including pharmacokinetic analysis with these dosing regimens in the future may allow for further optimization of the experimental design to rule out synergy or additivity between MMV048, PQ, and CQ. Ideally only one control group will be needed for future studies.

In this model, we observed *P. vivax* relapse to occur asynchronously and at all latent time points sampled. The studies were not designed to understand if relapse at certain time intervals is more common as observed in the field for parasites originating from different geographic regions. The isolates we used originated from Thailand, which is consistent with tropical strain relapse frequency at an average of 3 weeks post-infection in the presence of fast-clearing blood-stage antimalarials.[59] Further experiments will need to be designed to understand if the large sporozoite dose used in our model system leads to a rapid succession of activation events rather than discrete, apparently synchronized events.

A pre-clinical, small animal model for *P. vivax* drug development is lacking. While the *P. cynomolgi* nonhuman primate model can be used to assess the activity of pre-clinical drug candidates, it cannot assess the activity of drugs directly on *P. vivax* parasites. We present the establishment of a model that demonstrates anti-activation efficacy of primaquine on hypnozoites. Further studies are needed to establish the minimum inoculum and dosing regimen for controls in the model. The FRG huHep system we describe here could bridge the gap between *in vitro* drug discovery and pre-clinical studies in non-human primates and as such could accelerate the entry of candidate drugs into clinical development for radical cure of *P. vivax*.

MATERIALS AND METHODS

P. vivax sporozoite production

Anopheles dirus mosquitoes were infected with blood collected from *P. vivax* infected patients as described previously.[29] In brief, 2 mL of whole blood was washed once with RPMI160 incomplete medium. Packed infected blood was resuspended with and equal volume of pooled naive human AB serum to make 50% hematocrit. The suspension was fed for 30 min to 400 female *An. dirus* via an artificial membrane attached to a water-jacketed glass feeder maintained at 37°C. The engorged mosquitoes were maintained on a 10% sucrose solution and incubated at 26°C and 80% humidity for at least 14 days. Salivary gland dissections for harvesting sporozoites were performed at days 14–19 post feeding.

FRG huHep infection, liver extraction and pre-processing

All animal procedures were conducted in accordance with and approved by the Center for Infectious Disease Research Institutional Animal Care and Use Committee. The Center for the Infectious Disease Research adheres to the NIH Office of Laboratory Animal Welfare standards (OLAW welfare assurance #A3640-01). FRG huHep mice were infected intravenously through the tail vein or via retroorbital injection with sporozoites isolated from the salivary glands of infected mosquitoes. Sporozoites inocula were suspended in 100 μL of RPMI media. Mice were also infected by the bite of 20 infectious mosquitoes.[60] Mice were euthanized and livers were perfused with PBS through the hepatic portal vein, removed, and separated into lobes. Each lobe was cut into two sections with one reserved for IFA (immunofluorescence assay) and the other reserved for RNA extraction and qPCR. Lobes used for microscopy were fixed in 4% electron microscopy grade formaldehyde in PBS, which was replaced after 24 h with TBS + 0.05% sodium azide. The liver lobes were subsequently stored at 4°C in TBS containing 0.05% sodium

azide. Lobes used for qPCR were pooled and added to TRIzol reagent (Invitrogen).

qPCR

Pooled liver lobes were homogenized in TRIzol reagent and RNA was extracted using the Rneasy purification kit (QIAGEN). Quantification of parasite liver burden by 18S rRNA qPCR was done as described previously[61] with modifications as follows due to the liver tissue presence: pan-*Plasmodium* 18S rRNA primers and a probe were as reported,[62] and results from the extracted, *in-vitro*-transcribed RNA standard curve were normalized to a duplexed reaction for human GAPDH mRNA to control for the percentage of human hepatocyte repopulation. Human GAPDH mRNA-specific reagents were the PrimeTime Predesigned qPCR Assay (HsPT.39a.22214836; IDT). The limit of quantification of the assay is 3 $\log_{10}$ 18S rRNA.[61]

IFA

IFAs were carried out as described previously.[30] In brief, individual liver lobes were mounted on microscope slides and sliced to 50 μM using a vibratome. Liver sections were permeabilized in 10% H_2O_2 and 0.25% Triton X-100 in 1× TBS for 30 min at room temperature with agitation then washed in 1× TBS and blocked in 5% milk in 1× TBS for 1 h at room temperature. The primary antibodies were diluted in 5% milk and liver sections were incubated overnight at 4°C. After primary antibody staining liver sections were washed five times before addition of secondary antibodies for 2 h at room temperature. All liver-stage counts were assessed using antibodies to UIS4 and HSP70 or ACP.[17] Liver sections were again washed and transferred to 1 mL 0.06% $KmnO_4$ in H_2O. After another wash, cells were stained in 2 μg/mL DAPI for 5 min at room temperature. After washing sections were mounted onto microscope slides using Pro-long Gold Antifade reagent to preserve fluorescence. A minimum of three or six liver sections were quantified per animal when evaluating the primary infection (3–10 days post-infection) or the latent infection (>10 days post-infection), respectively.

Statistical methods

All statistical methods were conducted using GraphPad Prism 6.0 for Mac. The number of animals per experiment are depicted by each data points in the graph or stated in the figure legends. Parametric statistical methods (t test, Pearson correlation, ANOVA with Tukey's post-hoc test for multiple comparisons) were used for qPCR data and non-parametric (Kruskal-Wallis, Spearman correlation) were used for parasite counts due to its non-normal distribution.

Data availability statement

All data and supporting materials are available within the article and supplemental information.

SUPPLEMENTAL INFORMATION

Supplemental information can be found online at https://doi.org/10.1016/j.omtm.2022.07.016.

ACKNOWLEDGMENTS

This work was supported by the Bill and Melinda Gates Foundation (grant OPP1137694 to J.S.) and the German Research Foundation (fellowship SCHA2047/1-1 to C.S.).

AUTHOR CONTRIBUTIONS

E.L.F. wrote the manuscript. S.H.I.K., S.A.M., J.S., S.C.M., and B.C. edited the manuscript and designed the experiments. N.K., V.C., W.R., M.F., K.Z., Z.P.B., T.M., T.M.O., and C.S. performed the experiments.

DECLARATION OF INTERESTS

E.L.F., V.C., M.F., and S.A.M. are employed by and/or shareholder of Novartis Pharma AG.

REFERENCES

1. Taylor, A.R., Watson, J.A., Chu, C.S., Puaprasert, K., Duanguppama, J., Day, N.P.J., Nosten, F., Neafsey, D.E., Buckee, C.O., Imwong, M., and White, N.J. (2019). Resolving the cause of recurrent Plasmodium vivax malaria probabilistically. Nat. Commun. *10*, 5595–5611.
2. Robinson, L.J., Wampfler, R., Betuela, I., Karl, S., White, M.T., Li Wai Suen, C.S.N., Hofmann, N.E., Kinboro, B., Waltmann, A., Brewster, J., et al. (2015). Strategies for understanding and reducing the plasmodium vivax and plasmodium ovale hypnozoite reservoir in Papua New Guinean children: a randomised placebo-controlled trial and mathematical model. PLoS Med. *12*. e1001891–26.
3. Recht, J., Ashley, E., and White, N. (2014). Safety of 8-aminoquinoline Antimalarial Medicines (World Health Organization).
4. Howes, R.E., Dewi, M., Hogg, M.M., Battle, K.E., Padilla, C.D., Baird, J.K., and Hay, S.I. (2012). G6PD deficiency prevalence and estimates of affected populations in malaria endemic countries : A Geostatistical Model-Based Map. PLoS Med. *9*, 1–15.
5. Rabinovich, R.N., Drakeley, C., Djimde, A.A., Hall, B.F., Hay, S.I., Hemingway, J., Kaslow, D.C., Noor, A., Okumu, F., Steketee, R., et al. (2017). malERA: an updated research agenda for malaria elimination and eradication. PLoS Med. *14*. e1002456–17.
6. Wells, T.N.C., Burrows, J.N., and Baird, J.K. (2010). Targeting the hypnozoite reservoir of Plasmodium vivax : the hidden obstacle to malaria elimination. Trends Parasitol. *26*, 145–151.
7. Cogswell, F.B. (1992). The hypnozoite and relapse in primate malaria. Clin. Microbiol. Rev. *5*, 26–35.
8. Krotoski, W.A., Collins, W.E., Bray, R.S., Garnham, P.C., Cogswell, F.B., Gwadz, R.W., Killick-Kendrick, R., Wolf, R., Sinden, R., Koontz, L.C., and Stanfill, P.S. (1982). Demonstration of hypnozoites in sporozoite-transmitted plasmodium vivax infection. Am. J. Trop. Med. Hyg. *31*, 1291–1293.
9. Sattabongkot, J., Yimamnuaychoke, N., Leelaudomlipi, S., Rasameesoraj, M., Jenwithisuk, R., Coleman, R.E., Udomsangpetch, R., Cui, L., and Brewer, T.G. (2006). Establishment of a human hepatocyte line that supports in vitro development of the exo-erythrocytic stages of the malaria parasites Plasmodium falciparum and P. vivax. Am. J. Trop. Med. Hyg. *74*, 708–715.
10. Dembele, L., Gego, A., Zeeman, A.-M., Franetich, J.-F., Silvie, O., Rametti, A., Le Grand, R., Dereuddre-Bosquet, N., Sauerwein, R., van Gemert, G.-J., et al. (2011). Towards an in vitro model of Plasmodium hypnozoites suitable for drug discovery. PLoS One *6*. e18162–7.
11. Hollingdale, M.R., Collins, W.E., Campbell, C.C., and Schwartz, A.L. (1985). In vitro culture of two populations (dividing and nondivid) of exoerythrocytic parasites of Plasmodium vivax. Am. J. Trop. Med. Hyg. *34*, 216–222.
12. Roth, A., Maher, S.P., Conway, A.J., Ubalee, R., Chaumeau, V., Andolina, C., Kaba, S.A., Vantaux, A., Bakowski, M.A., Thomson-Luque, R., et al. (2018). A comprehensive model for assessment of liver stage therapies targeting Plasmodium vivax and Plasmodium falciparum. Nat. Commun. *9*, 2317–2416.

13. Gural, N., Mancio-Silva, L., Miller, A.B., Galstian, A., Butty, V.L., Levine, S.S., Patrapuvich, R., Desai, S.P., Mikolajczak, S.A., Kappe, S.H.I., et al. (2018). In vitro culture, drug sensitivity, and transcriptome of plasmodium vivax hypnozoites. Cell Host Microbe *23*, 395–406.e4.

14. Dembélé, L., Franetich, J.-F., Lorthiois, A., Gego, A., Zeeman, A.-M., Kocken, C.H.M., Le Grand, R., Dereuddre-Bosquet, N., van Gemert, G.-J., Sauerwein, R., et al. (2014). Persistence and activation of malaria hypnozoites in long-term primary hepatocyte cultures. Nat. Med. *20*, 307–312.

15. Zeeman, A.-M., van Amsterdam, S.M., McNamara, C.W., Voorberg-van der Wel, A., Klooster, E.J., van den Berg, A., Remarque, E.J., Plouffe, D.M., van Gemert, G.-J., Luty, A., et al. (2014). KAI407, a potent non-8-aminoquinoline compound that kills Plasmodium cynomolgi early dormant liver stage parasites in vitro. Antimicrob. Agents Chemother. *58*, 1586–1595.

16. Voorberg-van der Wel, A.M., Zeeman, A.M., Nieuwenhuis, I.G., van der Werff, N.M., Klooster, E.J., Klop, O., Vermaat, L.C., Kumar Gupta, D., Dembele, L., Diagana, T.T., et al. (2020). A dual fluorescent Plasmodium cynomolgi reporter line reveals in vitro malaria hypnozoite reactivation. Commun. Biol. *3*, 1–9.

17. Mikolajczak, S.A., Vaughan, A.M., Kangwanrangsan, N., Roobsoong, W., Fishbaugher, M., Yimamnuaychok, N., Rezakhani, N., Lakshmanan, V., Singh, N., Kaushansky, A., et al. (2015). Plasmodium vivax liver stage development and hypnozoite persistence in human liver-chimeric mice. Cell Host Microbe *17*, 526–535.

18. Azuma, H., Paulk, N., Ranade, A., Dorrell, C., Al-Dhalimy, M., Ellis, E., Strom, S., Kay, M. a, Finegold, M., and Grompe, M. (2007). Robust expansion of human hepatocytes in Fah-/-/Rag2-/-/Il2rg-/- mice. Nat. Biotechnol. *25*, 903–910.

19. Bissig, K.-D., Le, T.T., Woods, N.-B., and Verma, I.M. (2007). Repopulation of adult and neonatal mice with human hepatocytes: a chimeric animal model. Proc. Natl. Acad. Sci. USA *104*, 20507–20511.

20. Winzeler, E.A. (2018). A sleeping area of malaria research awakes. Cell Host Microbe *23*, 292–295.

21. Schäfer, C., Zanghi, G., Vaughan, A.M., and Kappe, S.H.I. (2021). Plasmodium vivax latent liver stage infection and relapse: biological insights and new experimental tools. Annu. Rev. Microbiol. *75*, 87–106.

22. Meis, J.F., Verhave, J.P., Meuwissen, J.H., Jap, P.H., Princen, H.M., and Yap, S.H. (1984). Fine structure of exoerythrocytic forms in cultured primary rat hepatocytes. Cell. Biol. Int. Rep. *8*, 755–765.

23. Aikawa, M., Schwartz, A., Uni, S., Nussenzweig, R., and Hollingdale, M. (1984). Ultrastructure of in vitro cultured exoerythrocytic stage of plasmodium berghei in a hepatoma cell line. Am. J. Trop. Med. Hyg. *33*, 792–799.

24. Shanks, G.D., and White, N.J. (2013). The activation of vivax malaria hypnozoites by infectious diseases. Lancet. Infect. Dis. *13*, 900–906.

25. Baird, J.K., and Hoffman, S.L. (2004). Primaquine therapy for malaria. Clin. Infect. Dis. *39*, 1336–1345.

26. Llanos-Cuentas, A., Lacerda, M.V.G., Hien, T.T., Vélez, I.D., Namaik-larp, C., Chu, C.S., Villegas, M.F., Val, F., Monteiro, W.M., Brito, M.A.M., et al. (2019). Tafenoquine versus primaquine to prevent relapse of plasmodium vivax malaria. N. Engl. J. Med. *380*, 229–241.

27. Lacerda, M.V., Llanos-Cuentas, A., Krudsood, S., Lon, C., Saunders, D.L., Mohammed, R., Yilma, D., Batista Pereira, D., Espino, F.E., Mia, R.Z., et al. (2019). Single-dose tafenoquine to prevent relapse of plasmodium vivax malaria. N. Engl. J. Med. *380*, 215–228.

28. Zeeman, A.M., Lakshminarayana, S.B., van der Werff, N., Klooster, E.J., Voorberg-van der Wel, A., Kondreddi, R.R., Bodenreider, C., Simon, O., Sauerwein, R., Yeung, B.K.S., et al. (2016). PI4 kinase is a prophylactic but not radical curative target in plasmodium vivax-type malaria parasites. Antimicrob. Agents Chemother. *60*, 2858–2863.

29. Sattabongkot, J., Kumpitak, C., and Kiattibutr, K. (2015). Membrane feeding assay to determine the infectiousness of Plasmodium vivax gametocytes. Methods. Mol. Biol. *1325*, 93–99.

30. Schafer, C., Dambrauskas, N., Steel, R.W., Carbonetti, S., Chuenchob, V., Flannery, E.L., Vigdorovich, V., Oliver, B.G., Roobsoong, W., Maher, S.P., et al. (2018). A recombinant antibody against Plasmodium vivax UIS4 for distinguishing replicating from dormant liver stages. Malar. J. *17*, 370–410.

31. Hodgson, S.H., Douglas, A.D., Edwards, N.J., Kimani, D., Elias, S.C., Chang, M., Daza, G., Seilie, A.M., Magiri, C., Muia, A., et al. (2015). Increased sample volume and use of quantitative reverse-transcription PCR can improve prediction of liver-to-blood inoculum size in controlled human malaria infection studies. Malar. J. *14*, 33–39.

32. Vanderberg, J.P. (1975). Development of infectivity by the plasmodium berghei sporozoite. J. Parasitol. *61*, 43–50.

33. Lindner, S.E., Swearingen, K.E., Shears, M.J., Walker, M.P., Vrana, E.N., Hart, K.J., Minns, A.M., Sinnis, P., Moritz, R.L., and Kappe, S.H.I. (2019). Transcriptomics and proteomics reveal two waves of translational repression during the maturation of malaria parasite sporozoites. Nat. Commun. *10*, 4964–5013.

34. Paquet, T., Le Manach, C., Cabrera, D.G., Younis, Y., Henrich, P.P., Abraham, T.S., Lee, M.C.S., Basak, R., Ghidelli-Disse, S., Lafuente-Monasterio, M.J., et al. (2017). Antimalarial efficacy of MMV390048, an inhibitor of Plasmodium phosphatidylinositol 4-kinase. Sci. Transl. Med. *9*. eaad9735–14.

35. McNamara, C.W., Lee, M.C., Lim, C.S., Lim, S.H., Roland, J., Simon, O., Yeung, B.K., Chatterjee, A.K., McCormack, S.L., Manary, M.J., et al. (2013). Targeting Plasmodium PI(4)K to eliminate malaria. Nature *504*, 248–253.

36. Gupta, D.K., Dembele, L., Voorberg-van der Wel, A., Roma, G., Yip, A., Chuenchob, V., Kangwanrangsan, N., Ishino, T., Vaughan, A.M., Kappe, S.H., et al. (2019). The Plasmodium liver-specific protein 2 (LISP2) is an early marker of liver stage development. Elife *8*. e43362–21.

37. Bertschi, N.L., Voorberg-van der Wel, A., Zeeman, A.-M., Schuierer, S., Nigsch, F., Carbone, W., Knehr, J., Gupta, D.K., Hofman, S.O., van der Werff, N., et al. (2018). Transcriptomic analysis reveals reduced transcriptional activity in the malaria parasite Plasmodium cynomolgi during progression into dormancy. Elife *7*. e41081–17.

38. Jones, R., Craige, B., Alving, A.S., Whorton, C.M., Pullman, T.N., and Eichelberger, L. (1948). A study of the prophylactic effectiveness of several 8-aminoquinolines in sporozoite-induced vivax malaria (Chesson strain). J. Clin. Invest. *27*, 6–11.

39. Arnold, J., Alving, A.S., Hockwald, R.S., Clayman, C.B., Dern, R.J., Beutler, E., Flanagan, C.L., and Jeffery, G.M. (1955). The Antimalarial action of Primaquine against the blood and tissue stages of falciparum malaria. J. Lab. Clin. Med. *46*, 391–397.

40. Flannery, E.L., Foquet, L., Chuenchob, V., Fishbaugher, M., Billman, Z., Navarro, M.J., Betz, W., Olsen, T.M., Lee, J., Camargo, N., et al. (2018). Assessing drug efficacy against Plasmodium falciparum liver stages in vivo. JCI Insight *3*, 92587–92612.

41. Shute, P.G., Lupascu, G., Branzei, P., Maryon, M., Constantinescu, P., Bruce-Chwatt, L.J., Draper, C.C., Killick-Kendrick, R., and Garnham, P.C. (1976). A strain of Plasmodium vivax characterized by prolonged incubation: the effect of numbers of sporozoites on the length of the prepatent period. Trans. R. Soc. Trop. Med. Hyg. *70*, 474–481.

42. Ungureanu, E., Killick-Kendrick, R., Garnham, P.C., Branzei, P., Romanescu, C., and Shute, P.G. (1976). Prepatent periods of a tropical strain of Plasmodium vivax after inoculations of tenfold dilutions of sporozoites. Trans. R. Soc. Trop. Med. Hyg. *70*, 482–483.

43. Minassian, A.M., Themistocleous, Y., Silk, S.E., Barrett, J.R., Kemp, A., Quinkert, D., Nielsen, C.M., Edwards, N.J., Rawlinson, T.A., Ramos Lopez, F., et al. (2021). Controlled human malaria infection with a clone of Plasmodium vivax with high-quality genome assembly. JCI Insight *6*, 1–18.

44. Sahu, T., Lambert, L., Herrod, J., Conteh, S., Orr-Gonzalez, S., Carter, D., and Duffy, P.E. (2015). Chloroquine neither eliminates liver stage parasites nor delays their development in a murine Chemoprophylaxis Vaccination model. Front. Microbiol. *6*, 1–6.

45. Douglas, A.D., Edwards, N.J., Duncan, C.J.A., Thompson, F.M., Sheehy, S.H., O'Hara, G.A., Anagnostou, N., Walther, M., Webster, D.P., Dunachie, S.J., et al. (2013). Comparison of modeling methods to determine liver-to-blood inocula and parasite multiplication rates during controlled human malaria infection. J. Infect. Dis. *208*, 340–345.

46. Adekunle, A.I., Pinkevych, M., McGready, R., Luxemburger, C., White, L.J., Nosten, F., Cromer, D., and Davenport, M.P. (2015). Modeling the dynamics of plasmodium vivax infection and hypnozoite reactivation in vivo. PLoS Neglected Trop. Dis. *9*. e0003595–18.

47. White, M.T., Karl, S., Battle, K.E., Hay, S.I., Mueller, I., and Ghani, A.C. (2014). Modelling the contribution of the hypnozoite reservoir to Plasmodium vivax transmission. Elife *3*, 1–19.

48. Holmes, M.J., Augusto, L.d.S., Zhang, M., Wek, R.C., and Sullivan, W.J. (2017). Translational control in the latency of apicomplexan parasites. Trends Parasitol. *33*, 947–960.

49. White, N.J. (2021). Anti-malarial drug effects on parasite dynamics in vivax malaria. Malar. J. *20*, 161–212.

50. Schmidt, L.H. (1986). Compatibility of relapse patterns of P cynomolgy infections in rhesus monkeys with continuous clinical development and hypnozoite concepts of relapse. Am. J. Trop. Med. Hyg. *35*, 1077–1099.

51. Meltzer, E., Rahav, G., and Schwartz, E. (2018). Vivax malaria chemoprophylaxis: the role of atovaquone-proguanil compared to other options. Clin. Infect. Dis. *66*, 1751–1755.

52. Camarda, G., Jirawatcharadech, P., Priestley, R.S., Saif, A., March, S., Wong, M.H.L., Leung, S., Miller, A.B., Baker, D.A., Alano, P., et al. (2019). Antimalarial activity of primaquine operates via a two-step biochemical relay. Nat. Commun. *10*, 3226–3229.

53. Boulard, Y., Landau, I., Miltgen, F., Ellis, D.S., Peters, W., Landau, I., Miltgen, F., Ellis, D.S., and The, W.P. (1983). The chemotherapy of rodent malaria, XXXIV. Causal prophylaxis Part III: ultrastructural changes induced in exo-erythrocytic schizonts of Plasmodium yoelii yoelii by primaquine. Ann. Trop. Med. Parasitol. *77*, 555–568.

54. White, N.J. (2013). Primaquine to prevent transmission of falciparum malaria. Lancet. Infect. Dis. *13*, 175–181.

55. Bennett, J.W., Pybus, B.S., Yadava, A., Tosh, D., Sousa, J.C., McCarthy, W.F., Deye, G., Melendez, V., and Ockenhouse, C.F. (2013). Primaquine failure and cytochrome P-450 2D6 in plasmodium vivax malaria. N. Engl. J. Med. *369*, 1381–1382.

56. Llanos-Cuentas, A., Lacerda, M.V., Rueangweerayut, R., Krudsood, S., Gupta, S.K., Kochar, S.K., Arthur, P., Chuenchom, N., Möhrle, J.J., Duparc, S., et al. (2014). Tafenoquine plus chloroquine for the treatment and relapse prevention of Plasmodium vivax malaria (DETECTIVE): a multicentre, double-blind, randomised, phase 2b dose-selection study. Lancet *383*, 1049–1058.

57. Payne, R.O., Griffin, P.M., McCarthy, J.S., and Draper, S.J. (2017). Plasmodium vivax controlled human malaria infection – progress and prospects. Trends Parasitol. *33*, 141–150.

58. Bennett, J.W., Yadava, A., Tosh, D., Sattabongkot, J., Komisar, J., Ware, L.A., McCarthy, W.F., Cowden, J.J., Regules, J., Spring, M.D., et al. (2016). Phase 1/2a trial of plasmodium vivax malaria vaccine candidate VMP001/AS01B in malaria-naive adults: safety, immunogenicity, and efficacy. PLoS Neglected Trop. Dis. *10*. e0004423–16.

59. White, N.J. (2011). Determinants of relapse periodicity in Plasmodium vivax malaria. Malar. J. *10*, 297.

60. Sack, B.K., Mikolajczak, S.A., Fishbaugher, M., Vaughan, A.M., Flannery, E.L., Nguyen, T., Betz, W., Jane Navarro, M., Foquet, L., Steel, R.W.J., et al. (2017). Humoral protection against mosquito bite-transmitted Plasmodium falciparum infection in humanized mice. Npj Vaccines *2*, 1–11.

61. Murphy, S.C., Daza, G., Chang, M., and Coombs, R. (2012). Laser cutting eliminates nucleic acid cross-contamination in dried-blood-spot processing. J. Clin. Microbiol. *50*, 4128–4130.

62. Billman, Z.P., Seilie, A.M., and Murphy, S.C. (2016). Purification of plasmodium sporozoites enhances parasite-specific CD8+ T cell responses. Infect. Immun. *84*, 2233–2242.

OMTM, Volume *26*

Supplemental information

Plasmodium vivax latent liver infection is characterized by persistent hypnozoites, hypnozoite-derived schizonts, and time-dependent efficacy of primaquine

Erika L. Flannery, Niwat Kangwanrangsan, Vorada Chuenchob, Wanlapa Roobsoong, Matthew Fishbaugher, Kevin Zhou, Zachary P. Billman, Thomas Martinson, Tayla M. Olsen, Carola Schäfer, Brice Campo, Sean C. Murphy, Sebastian A. Mikolajczak, Stefan H.I. Kappe, and Jetsumon Sattabongkot

Supplemental Material

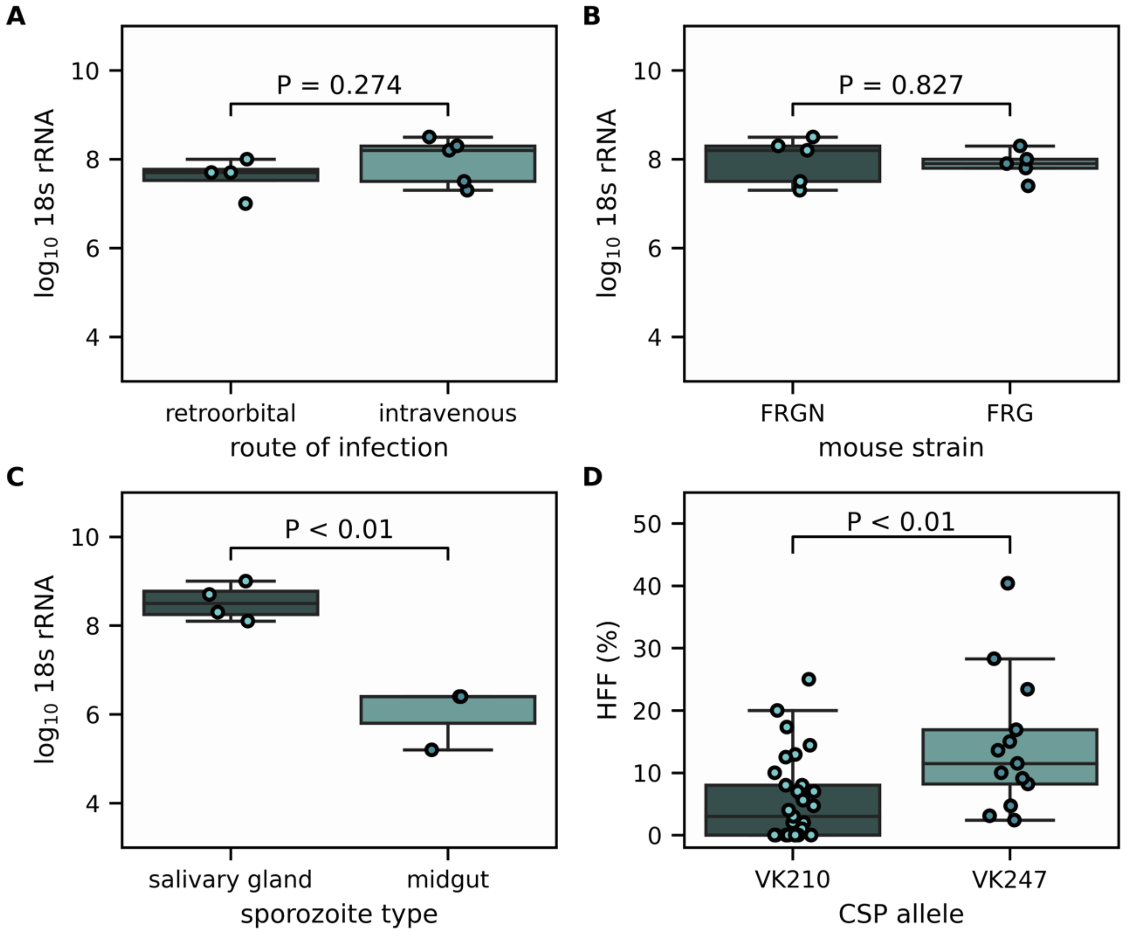

Figure S1. Additional characteristics of *P. vivax* liver-chimeric humanized mouse infections. (A) *P. vivax* 18S rRNA total liver burden 8 days after infection with 1.0 million sporozoites either intravenously or retro orbitally (p = 0.274, unpaired t-test). (B) *P. vivax* 18S rRNA total liver burden 8 days post-infection in FRGN (NOD) vs. FRG (C56Bl/6) mice infected with 1 million sporozoites (p = 0.827, unpaired t-test). (C) *P. vivax* 18S rRNA total liver burden 8 days post- infection with 0.6 million salivary gland- or midgut oocyst-dissected sporozoites (p < 0.01, unpaired t-test). (D) Hypnozoite formation frequency (HFF) of all isolates in Figure 1E-G (7 VK210 allele isolates and 3

VK247 allele isolates, the number of animals contributing from each isolate is denoted in Figure 1F, each dot represents one animal) by CSP allele type ($p < 0.01$, Mann Whitney test). In all figures, each data point represents observations from one animal. Parasite isolates used were (A, B): VUNL41, (C): VTTY111.

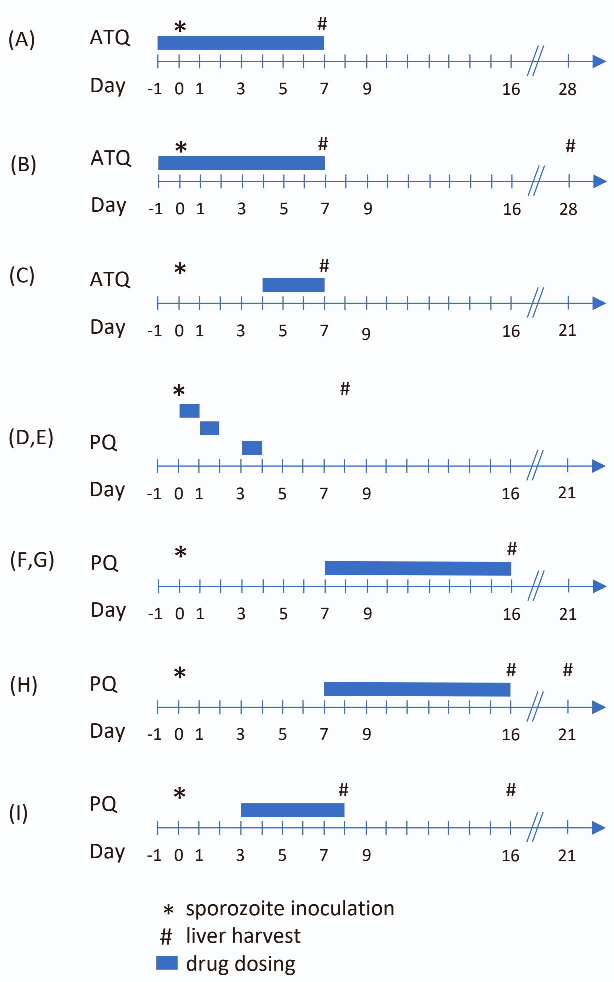

Figure S2. Dosing regimens for experiments with primaquine and atovaquone. FRG huHep mice were infected and treated at different timepoints with either atovaquone (ATQ) or primaquine (PQ). Results are presented in Figure 4.

1-11

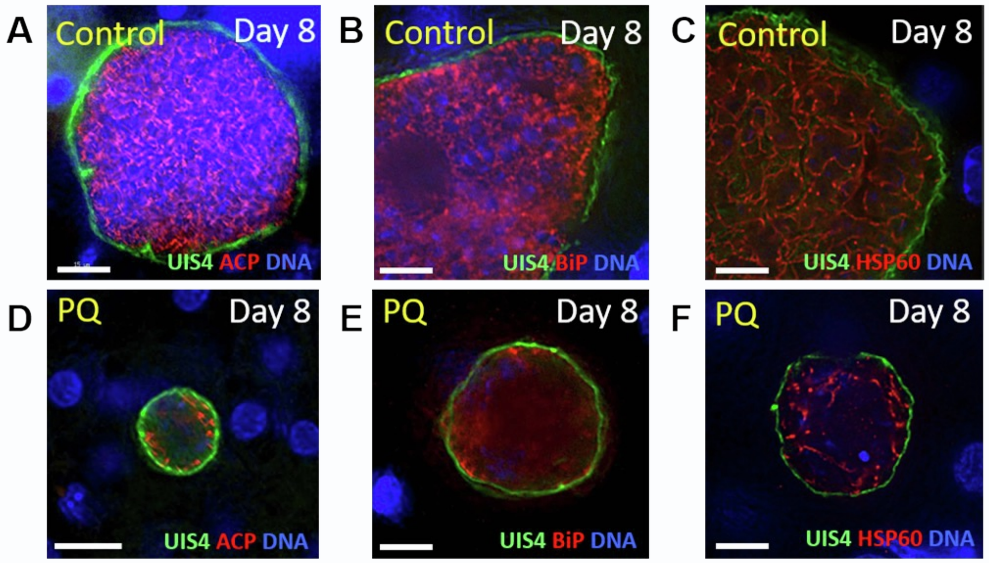

Figure S3. Effect of Primaquine treatment on *P. vivax* liver stage organelle development. FRG huHep mice infected with *P. vivax* sporozoites were untreated (Control) or treated with Primaquine (PQ) at day 3 and 4 and sacrificed at day 8 post inoculation. Immunofluorescence staining (IFA) was performed for (A,D) apicoplast (ACP – red), (B,E) ER (BiP – red), (C,F) mitochondria (HSP60 -red), parasitophorous vacuole membrane (UIS4 - green), and DNA (DAPI - blue); scale 10 μm.

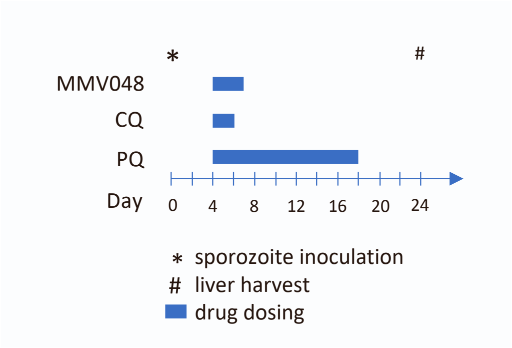

Figure S4. Dosing regimen used to demonstrate radical cure. FRG huHep mice were infected and livers were harvested 24 days later. Mice were either untreated, treated with MMV048 alone (30 mg/kg, days 4-7), PQ alone (30 mg/kg, days 4 -18), CQ alone (10 mg/kg, days 4-6) or in combination.
